# Genetic diversity and accession structure in European *Cynara cardunculus* collections

**DOI:** 10.1371/journal.pone.0178770

**Published:** 2017-06-01

**Authors:** Mario A. Pagnotta, Juan A. Fernández, Gabriella Sonnante, Catalina Egea-Gilabert

**Affiliations:** 1 Dipartimento di Scienze Agrarie e Forestali (DAFNE), Tuscia University, Viterbo, Italy; 2 Ciencia y Tecnología Agraria, E.T.S. Ingeniería Agrónoma, Universidad Politécnica de Cartagena, Cartagena (Murcia), Spain; 3 Producción vegetal, E.T.S. Ingeniería Agrónoma, Universidad Politécnica de Cartagena, Cartagena (Murcia), Spain; 4 Instituto de Biotecnología Vegetal (IBV), Universidad Politécnica de Cartagena, Cartagena (Murcia), Spain; 5 Institute of Biosciences and Bioresources, National Research Council (CNR), Bari, Italy; Università Politecnica delle Marche, ITALY

## Abstract

Understanding the distribution of genetic variations and accession structures is an important factor for managing genetic resources, but also for using proper germplasm in association map analyses and breeding programs. The globe artichoke is the fourth most important horticultural crop in Europe. Here, we report the results of a molecular analysis of a collection including globe artichoke and leafy cardoon germplasm present in the Italian, French and Spanish gene banks. The aims of this study were to: (i) assess the diversity present in European collections, (ii) determine the population structure, (iii) measure the genetic distance between accessions; (iv) cluster the accessions; (v) properly distinguish accessions present in the different national collections carrying the same name; and (vi) understand the diversity distribution in relation to the gene bank and the geographic origin of the germplasm. A total of 556 individuals grouped into 174 accessions of distinct typologies were analyzed by different types of molecular markers, i.e. dominant (ISSR and AFLP) and co-dominant (SSR). The data of the two crops (globe artichoke and leafy cardoon) were analyzed jointly and separately to compute, among other aims, the gene diversity, heterozygosity (He, Ho), fixation indexes, AMOVA, genetic distance and structure. The findings underline the huge diversity present in the analyzed material, and the existence of alleles that are able to discriminate among accessions. The accessions were clustered not only on the basis of their typology, but also on the basis of the gene bank they come from. Probably, the environmental conditions of the different field gene banks affected germplasm conservation. These outcomes will be useful in plant breeding to select accessions and to fingerprint varieties. Moreover, the results highlight the particular attention that should be paid to the method used to conserve the *Cynara cardunculus* germplasm and suggest to the preference of using accessions from different gene banks to run an association map.

## Introduction

*Cynara cardunculus* is a diploid species with 2n = 2x = 34 belonging to the Asteraceae family. A recent botanical classification grouped two vegetable crops and their wild progenitor, which were previously considered distinct species, under *C*. *cardunculus*. The three taxa within the species are: wild cardoon (*C*. *cardunculus* var. *sylvestris* (Lamk) Fiori), globe artichoke (*C*. *cardunculus* var. *scolymus* (L.) Fiori), and cultivated or leafy cardoon (*C*. *cardunculus* var. *altilis* DC). In line with this classification, the updated CPVO/UPOV protocol (entered into force on 27.02.2013) to tests on distinctness, uniformity and stability, has become the same for both the globe artichoke and the leafy cardoon [1].

Globe artichoke germplasm can be classified according to different criteria, the most important of which are (i) the harvesting time and (ii) the head shape. (i) The first divides the globe artichoke into (a) autumn and spring (early or “re-bloom” varieties) or (b) only spring (late or “spring” varieties). (ii) The second criterion classifies the globe artichoke on the basis of the morphology of its commercial part, the immature flowers (buds) called “capitula” or “heads”. In particular, the characteristics taken into consideration are: shape, color, and the presence of spines. It is therefore possible to identify four groups: "Spiny" with long sharp spines on both bracts and leaves, "Violet" with violet-colored capitula, "Romanesco" with spherical or sub-spherical non-spiny capitula, and "Catanese" with relatively small, elongated non-spiny capitula [2]. Spiny and Catanese types are normally re-blooming typologies, while Violet and Romanesco varieties are usually harvested in spring.

The globe artichoke is cultivated all around the world, but it is particularly well adapted to the different pedo-climatic conditions of the Mediterranean [3]. It is widely grown throughout the Mediterranean Basin, in South America (Chile, Argentina and Peru) and in California (USA). It is the fourth most important horticultural crop in Europe with a production of 815 kt, [4] after potato, tomato, and leafy vegetables. The leafy cardoon is a minor crop and is mainly grown in Northern Italy, Southern France and in Spain, for local consumption. The species is also important since it has nutraceutical, biochemical and medicinal properties (see [5] and references therein).

The center of origin of the globe artichoke is the Mediterranean Basin, probably southern Italy. Evidence based on its diversity and on morphological and molecular data indicates that the globe artichoke was possibly domesticated in Sicily at the beginning of the first millennium [6–8], while cardoon probably originated in the Iberian Peninsula and the South of France [8, 9]. In fact, Italy has the richest biodiversity of globe artichoke and cardoon, which has resulted in the local cultivation of many types of varieties and landraces, very often well adapted to specific local climatic conditions [5]. However, despite this wide biodiversity, the greatest part of Italian globe artichoke cultivation is based on very few clones [10].

The main *C*. *cardunculus* germplasm collections are held by Italy, France and Spain, where traditional globe artichoke cultivars are predominant. However, cultivations of single uniform varieties have expanded with a consequent reduction in diversity compared with the original. Indeed, in spite of the huge germplasm diversity available in Italy, only a few varieties are cultivated over large areas: ‘Violetto di Sicilia’, ‘Brindisino’, ‘Violet de Provence’ (all three belonging to the same Catanese group), ‘Romanesco’ (mainly the clone C3, now substituted by some clones derived from micro-propagation), and ‘Spinoso Sardo’. In France, only about five or six varieties are commonly cultivated. Almost all French production is based on the globe artichokes ‘Camus de Bretagne’, ‘Gros Vert de Laon’, ‘Blanc Hyerois’, ‘Violet du Gapeau’, ‘Castel’ and the small cylinder globe artichoke ‘Petit Violet de Provence’. In Spain, the ‘Blanca de Tudela’ ecotype represents 90% of the whole production.

In order to understand and preserve *C*. *cardunculus* genetic diversity it is important to know the propagation system, which differs between globe artichoke and cardoon. They are both allogamous plants, but the first is mainly propagated vegetatively by means of basal shoots or semi-dormant shoots with a limited root system [11], while the second is seed propagated. As a result, the level of heterozygosity in globe artichoke is higher than in cardoon, both wild and cultivated [5, 9, 12, 13]; moreover, the globe artichoke often has a multi-clonal structure. One problem in *C*. *cardunculus* conservation and characterization is that the local varieties are often named on the basis of the area where they are cultivated [14], regardless of their real genetic diversity or similarity. As a result, the names of accessions or local varieties are not always univocal and in some cases can generate synonymies or homonymies. In addition, previous studies have demonstrated that the huge diversity within each botanical variety is quite often not related with the corresponding geographic origin (e.g. [15–19]). Considering all this, it is clear that the characterization of *C*. *cardunculus* is essential for its correct conservation and utilization.

Moreover, it is sometime difficult to define globe artichoke varieties since, after cultivation over several decades in various geographical areas, they might be subjected to divergent selection. Thus, the accessions stored in gene bank field collections need to be rationalized by improving core collections and avoiding duplications [20–22].

Molecular markers are powerful tools that can be used to identify, cluster, and fingerprint individuals or accessions [23]. Several types of molecular markers have been used for cardoon and globe artichoke characterization in the last fifteen years. These include both dominant markers, which do not require *a priori* DNA sequence information, and co-dominant markers, for which sequence knowledge is necessary. The dominant markers include RAPD (Random Amplified Polymorphic DNA, [24–27]), AFLP (Amplified Fragment Length Polymorphism), and ISSR (Inter Simple Sequences Repeats), which were used with globe artichoke varieties and cardoon [28], or specific globe artichoke varietal groups, such as Spiny [29], Romanesco [15,30,31], Violet and Catanese [32–35], globe artichoke hybrids [36] and cardoon [37]. Among co-dominant markers, SSRs (Simple Sequence Repeats) [8, 34, 38–42] and SRAP (Sequence-Related Amplified Polymorphism) have been used for globe artichokes, and in both cultivated and wild cardoon [43].

The association between genotype and phenotype can be achieved either by controlled biparental crosses (linkage mapping) or via association mapping, controlling the linkage disequilibrium, i.e. the non-random association of alleles between loci, regardless of their position across the chromosomes [44]. The assessment of genetic variation and population structure is a prerequisite before performing association mapping. Linkage mapping has a series of limitations, including its high cost, low resolution, the need for polymorphism between the parents used, large segregant population and the distribution of chiasma across the genome. Conversely, an association map, using accessions that are not related by common parents, can detect several alleles at each locus and a higher level of polymorphism. Association mapping is a tool that can be used to investigate elite genes by structuring the natural variation present in a germplasm. Possible errors in association maps may arise due to unequal allele frequency distribution between subgroups, which may lead to spurious associations between molecular markers and the traits of interest [45]. In order to reduce such errors, before performing an association analysis in a population, it is essential to determine the population structure, as we do in this study.

Previous works on *C*. *cardunculus* characterization were addressed at specific accession typologies and used limited collections belonging to restricted geographic areas. The only exception is our previous paper, which reported preliminary data using only some of the dominant markers in the European collection [18]. The aims of the present paper are (i) to assess, for the first time, the diversity present in *C*. *cardunculus* European collections, using both dominant and co-dominant markers, (ii) to determine the population structure, (iii) to measure the genetic distance between accessions; and (iv) to cluster the accessions according to the molecular data. Moreover, two other questions are addressed: (v) are the accessions present in the different national collections and carrying the same name really the same material? and (vi) how is diversity distributed in relation to the geographic origin of the germplasm?

## Material and methods

### Plant material

A total of 556 individuals belonging to Italian (264) (CNR-IBBR, Bari; CNR ISAFOM Catania; ARSIAL Rome), French (162) (GEVES, Cavaillon) and Spanish (130) (ITGA, Navarra) collections, and representing 174 accessions were jointly analyzed. The accession list is reported in S1 Table together with their typology and country of conservation. The accessions are divided according to the four typologies described in the introduction and identified by Porceddu et al. [2]: Romanesco (225 individuals), Violet (34 individuals), Catanese (116 individuals) and Spiny (11 individuals), plus the accessions belonging to leafy cardoon (72 individuals). Moreover, two additional globe artichoke categories were added: the Blanca de Tudela typology (39 individuals) due to its importance in the Iberian Peninsula, and OFF (59 individuals), which includes the accessions not univocally classifiable as belonging to the previous typologies.

### Molecular markers

Total genomic DNA was extracted from 100 mg of frozen tissue, using the Qiagen DNeasy Plant Mini Kit and shared among laboratories to perform marker amplifications. For the ISSR markers, 11 primers were used: 810, 818, 827, 834, 840, 841, 855, 857, 857c, 857g and 872, all developed by the University of British Columbia, Canada. The reactions were performed in 10 μl containing 10 ng DNA, 0.3 μM primer, 100 μM dNTP, 10 mM Tris-HCl (pH 9.0) and 1U Taq polymerase. Amplification conditions were 94°C/5 min, followed by 35 cycles of 94°C/1 min, 43–59°C (specific for each primer; see S2 Table)/1min 60°C/1 min and 72°C/2 min, and ending with an extension step of 72°C/10 min.

For AFLP analysis, *Mse*I and *Pst*I or *Eco*RI and *Mse*I were used to digest the DNA template. Following the procedure described by Vos et al. [46], pre-amplification was performed with non-selective primers, while 7 primer combinations were used for the selective amplification step: EcoACC/MseCTA, EcoACG/MseCTT, EcoAGC/MseCTT, MseAC/PstCA, MseAC/PstCG, MseGC/PstCA and MseGC/PstCG.

For 19 SSR markers developed by Acquadro et al. [47] (CMAL06, CMAL-108, CMAL11, CMAL117, CMAL21, CMAL24, CMAL-25), Acquadro et al. [48], (CDAT-01, CLIB-02, CLIB-12), Acquadro et al. [40] (CMAFLP-01, CMAFLP-04, CMAFLP-05, CMAFLP-18), Sonnante et al. [39] (CsCiCaCa05, CsPal02, CsPal03, CsEST03) and FA2-GAT (Primers, F:GCCGAAGAAGACGGAAGAATCTGA, R:CATCACGCTTGGTTAAAGATCGGG) were used.

For all amplifications the forward primers were fluorescently labeled to resolve PCR amplicons on an ABI 3130xl (Applied Biosystems) or a CEQ 8800 (Beckman Coulter) sequencer. The detected bands were checked for reproducibility even if the visualization by sequencer showed high sensitivity and precision. For the co-dominant (SSR) markers, each allele was scored in accordance with its molecular weight in bp, while for the dominant (AFLP and ISSR) markers, 0–1 matrices were obtained, without knowing the allelic relationships. In this case, each possible band was considered as a locus with 2 possible alleles, 0 (absence) or 1 (band presence). In some cases, the 0–1 matrix was considered as haplotype, while in others it was converted into a co-dominant matrix with 1 as dominant homozygote (A/A) and 0 as the other homozygote (a/a).

### Statistical analyses

The gene diversity index was calculated for each locus and population according to Nei [49], using the Hardy-Weinberg formula

$$He=1-\sum_{i=1}^{n}p_{i}^{2}.$$

The polymorphism information content (PIC) was computed as [50].

$$PIC=1-\sum_{i=1}^{n}p_{i}^{2}-\sum_{i=1}^{n-1}\sum_{j=i+1}^{n}{2p}_{i}^{2}p_{j}^{2}$$

To compare differences among and within accessions and groups Wright’s fixation indices were used [51]. The *F*-statistics are based on the expected level of heterozygosity. The measurements were computed for the different levels of the accession structures, such as the variance of allele frequencies **within accessions** (*F*_*IS*_), variance of allele frequencies **among accessions** (*F*_*ST*_), inbreeding coefficient **within individual total diversity** (*F*_*IT*_), variance among accessions within types (*F*_*SC*_) and variance permuting accessions among groups (*F*_*CT*_), which are related to the degree of heterozygosity at various levels of the accession structure. The terms mentioned above are related through the formula: 1-F_IT_ = 1-F_IS_ + 1-F_ST_, where I indicates the individual, S the sub-accession and T the total accession; *F*_*IT*_ refers to the individual compared with the total; *F*_*IS*_ is the individual compared with the sub accession; and *F*_*ST*_ is the sub accession compared with the total. The total *F*, indicated by *F*_*IT*_, can be partitioned into *F*_*IS*_ (or *f*) and *F*_*ST*_ (or *θ*). *F*_*ST*_ can be computed using the formula: F_ST_ = (H_T_-H_S_)/H_T,_ where *H*_*T*_ is the proportion of the heterozygotes in full accessions and *H*_*S*_ the average proportion of heterozygotes in sub-accessions. The *F* statistic was also used in the AMOVA (Analysis of MOlecular Variance) to measure the partition of variation among typologies, among accessions within typologies, among individuals within accessions, and within individuals.

The genetic diversity (*He*) and genetic identity (*J* or *Ho*) were also used to estimate the genetic distance. If

$$J_{x}=\sum_{i=0}^{n}p_{xi}^{2}$$

is the probability of identity in the x accession and

$$J_{y}=\sum_{i=1}^{n}p_{yi}^{2}$$

is the probability of identity in the y accession, the probability of identity in both accessions is

$$J_{xy}=\sum_{i=1}^{n}p_{xi}p_{yi}$$

as described by Nei [52]. The probability of identity in the x accession for all normalized loci is

$$I=J_{xy}÷\sqrt{J_{x}J_{y}}$$

and, in turn, the genetic distance is:

$$D=-LnI-LnJ_{xy}÷\sqrt{J_{x}J_{y}}$$

The distances between accessions were also computed using the Euclidean distance

$$d=\sqrt{{\left(p_{1}p_{2}\right)}^{2}}$$

The obtained distance was then used to cluster the accessions according to different clustering methodologies such as the UPGMA algorithm. The clustering was also performed by K-mean, which is a non-hierarchical method of classification that partitions a set of samples into the most appropriate number of clusters decided in advance [53]. Run length, in STRUCTURE software, was given as a 150k burning period length followed by 150k Markov Chain Monte Carlo (MCMC) replications. As suggested, several analyses were first run using different K values from 2 to 9. Finally, in accordance with likelihood, ΔK [54] that identified two picks for K = 3 and K = 6 (S1 Fig), *F*_*ST*_ distribution among groups and with the fact that the germplasm could be divided into 6 groups on the basis of typology (Romanesco, Violet, Catanese, Spiny, Cardoon, and OFF), both K = 3 and K = 6 were adopted and are presented below. Individuals were assigned to subgroups by the “No admixture model”. The output reports the subsequent probability that individual *i* is from accession k. The prior probability for each accession is 1/K. This model is appropriate for studying fully discrete accessions. The “admixture model” was also run, but no better resolution was observed with this model (data not shown). Linkage disequilibrium between loci, which measures the deviation from random association between alleles at different loci [55], and its significance (*P* values of χ^2^ with 1000 permutations), was also computed.

In some cases, the co-dominant and dominant data typologies were analyzed separately, since it was not possible to compute some parameters for the dominant markers. In addition, analyses were run both jointly and separately for globe artichoke and cardoon accessions.

To compute the above mentioned parameters, GenAlEx [56,57], Power Marker [58], Arlequin [59], and STRUCTURE v 2.3 [45] software were used.

## Results

### Genetic diversity

The identified co-dominant alleles were 147 in total, from the 556 individuals analyzed. For each co-dominant marker, the alleles ranged from 1 to 15 with an average of 7.4 alleles per marker (Table 1). Nevertheless, most of the alleles (about 62%) were rare, having a frequency lower than 5%; as a result, the major alleles had an overall average frequency of 70%. For the co-dominant markers, the major alleles for each marker had a frequency ranging from about 23% to around 87%, excluding the CMAFLP-05 marker, which was monomorphic. Some of the SSR alleles were specific for a single accession (Table 2). In total, 33 private alleles were found in 24 accessions. As expected, the frequency was higher and more constant in the dominant markers with only two alleles. In fact, average frequency of private alleles was 0.816 and 0.805 for AFLP and ISSR respectively versus 0.700 for SSR, while it ranged from 0.743 to 0.878 and from 0.232 to 0.869 for dominant and co-dominant markers respectively.

**Table 1:** Markers used to genotype 556 *Cynara cardunculus* individuals, with their genetic diversity parameters.

Marker	Alleles	PAF	He	PIC	Ho	Fis	Fit	Fst	F
**AFLP**									
EaccMcta		0.799	0.255	0.203					
EacgMctt		0.801	0.291	0.242					
EagcMctt		0.835	0.219	0.176					
MacPca		0.796	0.283	0.231					
MacPcg		0.820	0.257	0.213					
MgcPca		0.828	0.249	0.207					
MgcPcg		0.832	0.244	0.204					
**Mean**		0.816	0.257	0.211					
**ISSR**									
810		0.848	0.245	0.210					
818		0.781	0.310	0.250					
827		0.766	0.331	0.271					
834		0.884	0.203	0.181					
840		0.756	0.333	0.267					
841		0.852	0.231	0.197					
855		0.817	0.279	0.234					
857		0.878	0.201	0.175					
857c		0.757	0.341	0.277					
857g		0.780	0.321	0.263					
872		0.743	0.332	0.264					
**Mean**		0.805	0.284	0.235					
**SSR loci**									
CsCiCaCa05	10	0.829	0.306	0.298	0.243	-0.287	0.228	0.400	-0.252
CDAT-01	7	0.311	0.768	0.734	0.806	-0.930	-0.048	0.457	-0.923
CLIB-02I	7	0.232	0.825	0.800	0.391	-0.655	0.446	0.665	-0.635
CLIB-02II	4	0.869	0.236	0.223	0.049	0.079	0.817	0.801	0.110
CLIB-12	3	0.365	0.663	0.589	0.790	-0.977	-0.232	0.377	-0.956
CMAFLP-01	6	0.741	0.425	0.397	0.375	-0.850	0.154	0.543	-0.804
CMAFLP-04	13	0.632	0.554	0.516	0.123	-0.554	0.815	0.881	-0.507
CMAFLP-05	1	1.000	0.000	0.000	0.000		1.000	1.000	
CMAFLP-18	12	0.500	0.605	0.532	0.750	-0.894	-0.301	0.313	-0.895
CMAL06	13	0.474	0.633	0.568	0.532	-0.749	0.050	0.457	-0.723
CMAL-108	4	0.561	0.582	0.513	0.628	-0.958	-0.090	0.444	-0.936
CMAL11	3	0.619	0.473	0.363	0.478	-0.612	-0.024	0.365	-0.555
CMAL117	14	0.737	0.415	0.371	0.240	-0.407	0.307	0.507	-0.336
CMAL21	10	0.632	0.553	0.515	0.527	-0.732	-0.024	0.409	-0.705
CMAL24	6	0.366	0.683	0.617	0.164	-0.511	0.649	0.767	-0.423
CMAL-25	2	0.504	0.500	0.375	0.992	-0.995	-0.925	0.035	-0.994
CsPal02	15	0.604	0.562	0.508	0.317	-0.476	0.372	0.575	-0.432
CsPal03	5	0.512	0.637	0.580	0.723	-0.851	-0.189	0.358	-0.836
CsEST03	8	0.666	0.484	0.418	0.403	-0.314	0.124	0.333	-0.289
FA2-GAT	4	0.864	0.236	0.209	0.244	-0.764	-0.024	0.419	-0.696
**Mean**	7.4	0.601	0.507	0.456	0.439	-0.655	0.155	0.505	-0.620
Overall mean		0.700	0.397	0.347					

Number of alleles in the case of SSR (Alleles), frequency of the principal allele (PAF), Gene diversity and Expected Heterozygosity (He), polymorphism information content (PIC), observed heterozygosity (Ho), variance of allele frequencies within accessions (Fis), inbreeding coefficient within an individual total diversity (Fit), variance of allele frequencies among accessions (Fst), Wright’s fixation indices (F).

**Table 2:** Summary of private alleles by accession.

Accession	Locus	Allele	Frequency
Ascolano	CMAFLP-04	262	0.313
BlancHyérois	CLIB-02I	221	0.333
BlancoPeralta	CMAFLP-04	253	1.000
BlancoValencia	EST03	195	0.100
BlancoValencia	EST03	198	0.100
Brindisi	CLIB-02II	223	0.500
Camard	FA2-GAT	187	0.100
CamusBretagneI	FA2-GAT	223	0.250
Caribou	CMAL117	168	0.100
Caribou	CMAFLP-04	272	0.125
CaribouSp	EST03	183	0.250
Chrysanthème	CMAL117	192	0.100
Chrysanthème	CsPal03	331	0.100
DelCortijo	EST03	201	0.100
DelCortijo	EST03	204	0.100
Mola	CMAL06	137	0.038
MonteluponeA	CsPal02	381	0.250
Paestum	CsPal02	343	0.063
Pertosa	CsPal02	355	0.125
Pisa	CMAL117	174	0.063
PuvisAmélioré	CMAL-108	101	0.167
PuvisAmélioré	CMAFLP-04	286	0.375
SErasmo	CMAL117	170	0.250
VerdeCalahorra	CMAL06	157	0.125
VerdePeralta	CsPal02	361	0.400
VertVaulxVelin	CMAL06	147	0.750
VertVaulxVelin	CsPal02	349	1.000
VertVaulxVelin	CMAFLP-01	224	0.167
VertVaulxVelin	CMAFLP-01	323	0.333
VertVaulxVelin	CMAFLP-04	273	0.250
ViolettoSicilia13	CMAL06	140	0.250
ViolettoSicilia98	CMAL11	275	0.250
ViolProvence41S	CMAL24	236	0.500

The gene diversity computed as expected heterozygosity (He) and the polymorphism information content (PIC) provide information of a marker’s ability to determine polymorphism. In the present study, He values for the dominant markers were quite uniform, with an average of around 0.26 and 0.28 for AFLP and ISSR, respectively (Table 1). For SSR, the gene diversity ranged from 0.24 (FA2-GAT and CLIB-02II) to 0.83 (CLIB-02I) (Table 1). Note that the marker CLIB-02 identified 2 loci, here labeled as CLIB-02I and CLIB-02II, so that CLIB-02 altogether had higher values. The PIC values had a similar but not equal ranking among markers compared with the gene diversity parameter. PIC ranged from 0.21 of FA2-GAT to 0.80 of CLIB-02I, followed by CDAT-01 with 0.73 (Table 1). The values were, on average, lower in leafy cardoon than in globe artichoke (S3 and S4 Tables).

For the co-dominant markers, it is also possible to compute the observed heterozygosity (Ho) and the Wright fixation indices by considering both He and Ho. The observed heterozygosity ranged from 5% for CLIB-02II to 99% for CMAL-25, with an average of 44% (Table 1). As a consequence, the partition of variation, into its components i.e. within accessions (*F*_*IS*_), within individuals (*F*_*IT*_), and among accessions (*F*_*ST*_) was quite different from one marker to another. The average values for the *F*_*IS*_, *F*_*IT*_ and *F*_*ST*_ were -0.66, 0.16 and 0.51, respectively (Table 1). Generally, the values were lower in leafy cardoon than in globe artichoke (S3 and S4 Tables). In general, the loci were in LD with each other (S5 Table) except for CMAL-25, which was not in LD with CMAL11, CMAL117, CMAL24, CsCaCa05, CsEST03 and CsPal02.

The accessions analyzed had quite different levels of diversity, as detected using both dominant and co-dominant markers, as shown in S6 and S7 Tables. The polymorphism ranged from 0 to about 55%, with an average of 20%, for the dominant markers and from 10 to 85%, with an average of 54%, for the co-dominant markers. The expected heterozygosity ranged from 0 to 17%, with an average of 7%, for dominant and from 5 to 42%, with an average of 26%, for co-dominant markers. The observed heterozygosity ranged from 10 to 67%, with an average of 45%. The fixation index was mainly negative, ranging from -1 to 0.24, with an average of -0.74. The accessions had on average more than one allele but, in spite of the high number of alleles identified by the markers, the alleles were generally specific for each accession. The alleles with a frequency higher than 5% in a single accession ranged from 0.6 to 2.3, and the number of locally common alleles found in less than 25% of the accessions but present in more than 5% in the specific accession ranged from 0 to 1.2, with an average of 0.2. Some accessions had more than a single private allele, reaching a maximum of five private alleles specific for VertVaulxVelin (Table 2). The Shannon Information Index indicated richness, and the evenness ranged from 0 to 0.26 for the dominant and from 0 to 0.66 for the co-dominant markers, with an average of 0.11 and 0.36, respectively (S6 Table). On average, the marker parameters for each accession ranged from 0.403 to 3.286 for the number of alleles; from 1 to 2.55 for the number of effective alleles, from 0 to 0.934 for the Shannon's Information index, from 0 to 0.517 for the expected heterozygosity; from 0 to 0.583 for the unbiased expected heterozygosity; and from 6 to 192 for the number of amplicons of the dominant markers (S7 Table).

### Accession structure and genetic relationships

**Fig 1:**
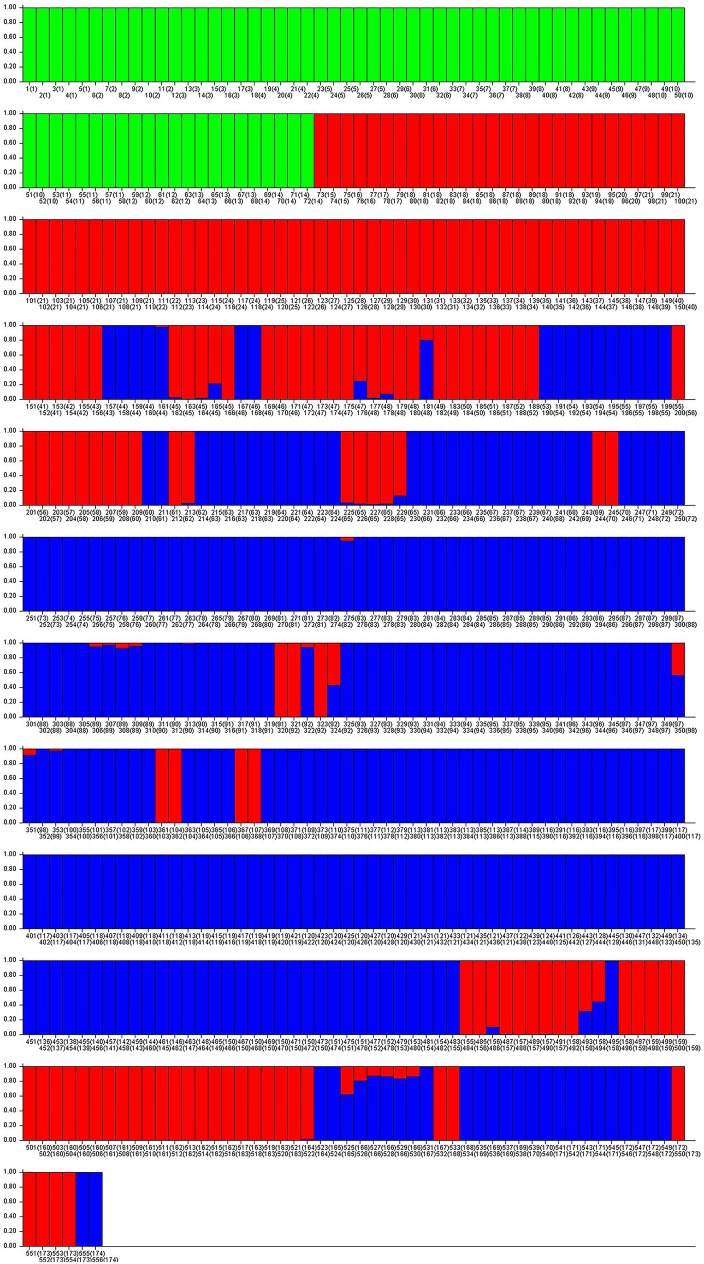
STRUCTURE analyses of 556 individuals based on 150˙000 permutations, No admixture model and K = 3. The accession order was: PleinBlancInerme, PuvisAmélioré, RougeAlger, VertVaulxVelin, BlancodeHuerva, BlancoPeralta, BlancoValencia, DelCortijo, LlenoEspaña, Lumbier, RojoAgreda, Sarramian, VerdeCalahorra, VerdePeralta, B1, B2, B7, Brindisino, Catanese, GagliardoSgrò, Mola, NiscemeseBA, NiscemeseCT, VioletProvenceI, ViolettoSicilia10, ViolettoSicilia13, ViolettoSicilia14, ViolettoSicilia3, ViolettoSicilia4, ViolettoSicilia61, ViolettoSicilia64, ViolettoSicilia98, ViolettoSiciliab3, ViolettoSiciliai2, ViolettoSiciliai3, ViolProvence41I, ViolProvence45I, ViolProvence73, ViolProvenceV, ViolProvenceVM, ViolProvenceVP, ViolProvenceVPG, ViolProvenceVR, Cacique, Chrysanthème, Escarot, VioletProvence45, VioletProvenceF, Brindisi, ChrysanthèmeS, Masedu, VioletProvence41S, Aquara, BiancoOstuni, Calimera, MC12, MC14, MC6, MO10, MO5, Motta, MT1, NeroCastrignano, NeroOstuni, Calice, GrosVertdeLaon, VertProvence, Carlit, France, Francesco, Italiana, BiancoPertosa, CamardI, CamerysI, CamusBretagneI, Capuanella, Isernia, Pietralcina, Pietrelcinab, RomanescoBA, RomanescoCT, TondoPaestum, Calico, Camard, Camus, Capitan, Caribou, Castel, Compact, Cric, Lira, Pètre, Popver, Romain, Salambo, Salanquet, Vertu, Apollo, C3, CalicoRojoCR, CalicoVerdeCB, CamerysS, CampagnanoS, CamusBretagneBH8, CamusBretagneS, CaribouSp, Macau, Moretto, MutRomanesco, Ñato, SalamboS, SalanquetS, Ascolano, Campagnano, GratoI, Jesino, MonteluponeA, MonteluponeB, Paestum, Pertosa, Pisa, S1, S10, S11, S13, S15, S16, S17, S18, S2, S20, S22, S23, S25, S26, S3, S30, S4, S5, S6, T31, T32, T33, T34, T35, T36, T37, T38, T39, TondoRossoPaestum, SpinosoPalermo, SpinosoViolLiguria, Criolla, Hysponos, SpinosoSardo, BlancHyèroisI, Blancal, BlancHyérois, Cabeza de gato, Clon303, INIA-B, INIA-D, ITGA, PAT89, SErasmo, TeromBA, TeromCT, VioletGapeauI, ViolettoMaremma, ViolettoToscana, Velours, VioletCamargue, VioletGapeau, Hydes.

**Fig 2:**
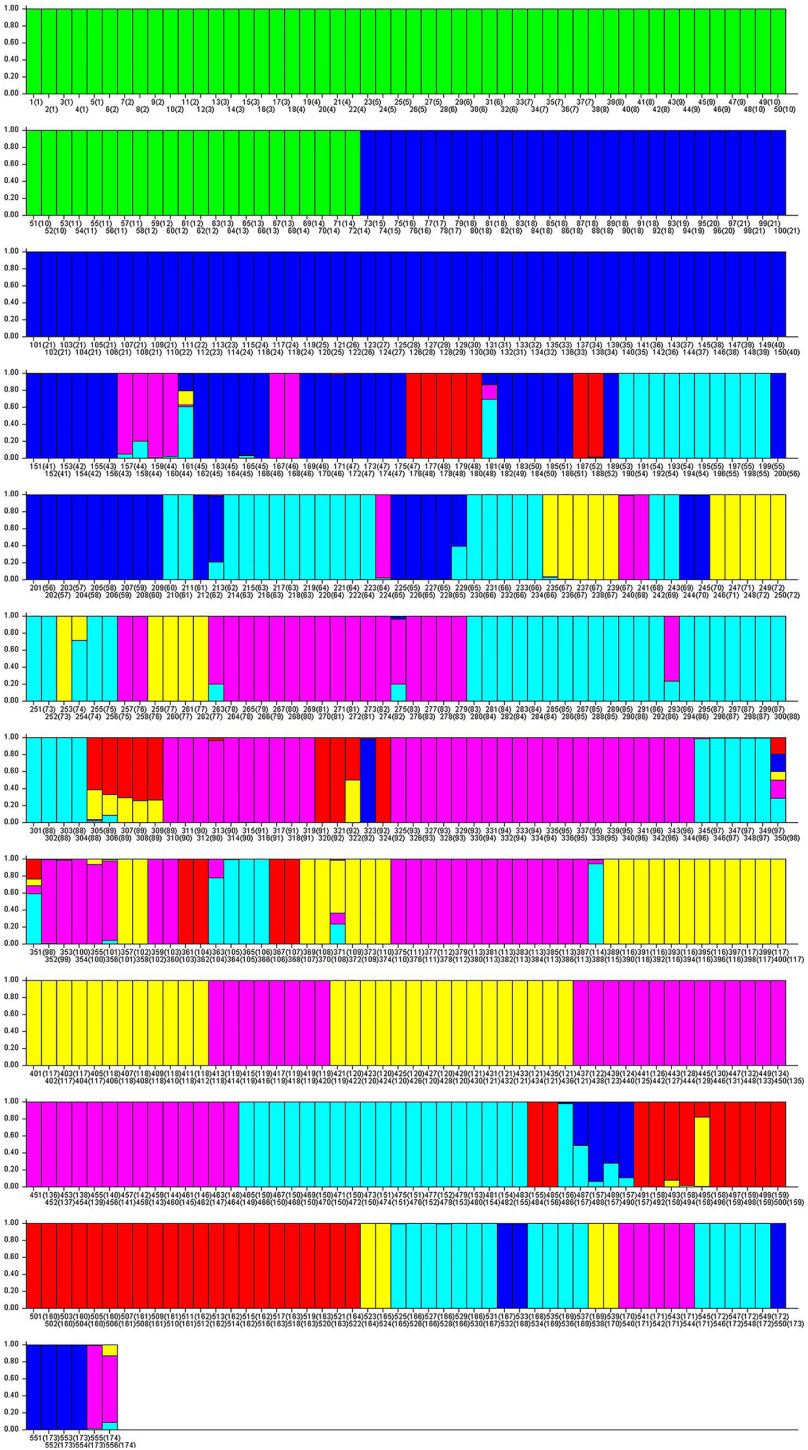
STRUCTURE analyses of 556 individuals based on 150˙000 permutations, No admixture model and K = 6. The accession order was: as in Fig 1.

The structure of the 174 accessions was analyzed by means of a Bayesian based approach in the STRUCTURE program, considering only the co-dominant loci. According to the Evanno [54] calculation, the most probable K was three (S1 Fig). The results obtained using STRUCTURE with K = 3 identified a first group with the leafy cardoon accessions, a second group with mainly the Catanese and Tudela accessions and a third group with all the other globe artichoke varieties (Fig 1). To better separate the non-Catanese globe artichokes, the structure analysis was repeated with K set equal to 6, which was the second most probable K in the Evanno analysis (S1 Fig). Fig 2 shows the Q value for each of the 556 individuals analyzed; the colored segments have lengths proportional to each of the K inferred clusters.

The first sub group (SG1), labeled in green in Fig 2, contained individuals from 1 to 72, which corresponded to the first 14 accessions, all of them belonging unequivocally to the leafy cardoon group. The second sub-group (SG2), labeled in blue, included most of the accessions of Catanese typology, but also some of the Tudela, some of the Violet, and some of the unassigned individuals such as Aquara, Francesco, MC12, MC14, MC6, MO10, MO5, and MT1. The third sub-group (SG3), labeled in turquoise, included all the Spiny, some Romanesco and some Violet individuals, and four unassigned (BiancoOstuni, Calimera, NeroCastrignano, and NeroOstuni). The fourth sub-group (SG4), labeled in purple, grouped most of the Romanesco types plus Hydes and Velours (Violet), Cacique (Catanese), and Carlit (unassigned). The fifth sub-group (SG5), labeled in yellow, contained 12 accessions of Romanesco types, two unassigned (Italiana and VertProvence), and two Violet types (ViolettoToscana and SErasmo). Finally, the sixth sub-group (SG6), labeled in red, included the Tudela accessions plus some Romanesco (Macau, CamusBretagneBH8) and Catanese (VioletProvenceF, VioletProvence41S). In the case of K equal 3 (Fig 1) the first group corresponded to SG1 including all the leafy cardoon accessions. The second group was similar to SG2 + SG6, while the third group included SG3, SG4 and SG5.

**Fig 3:**
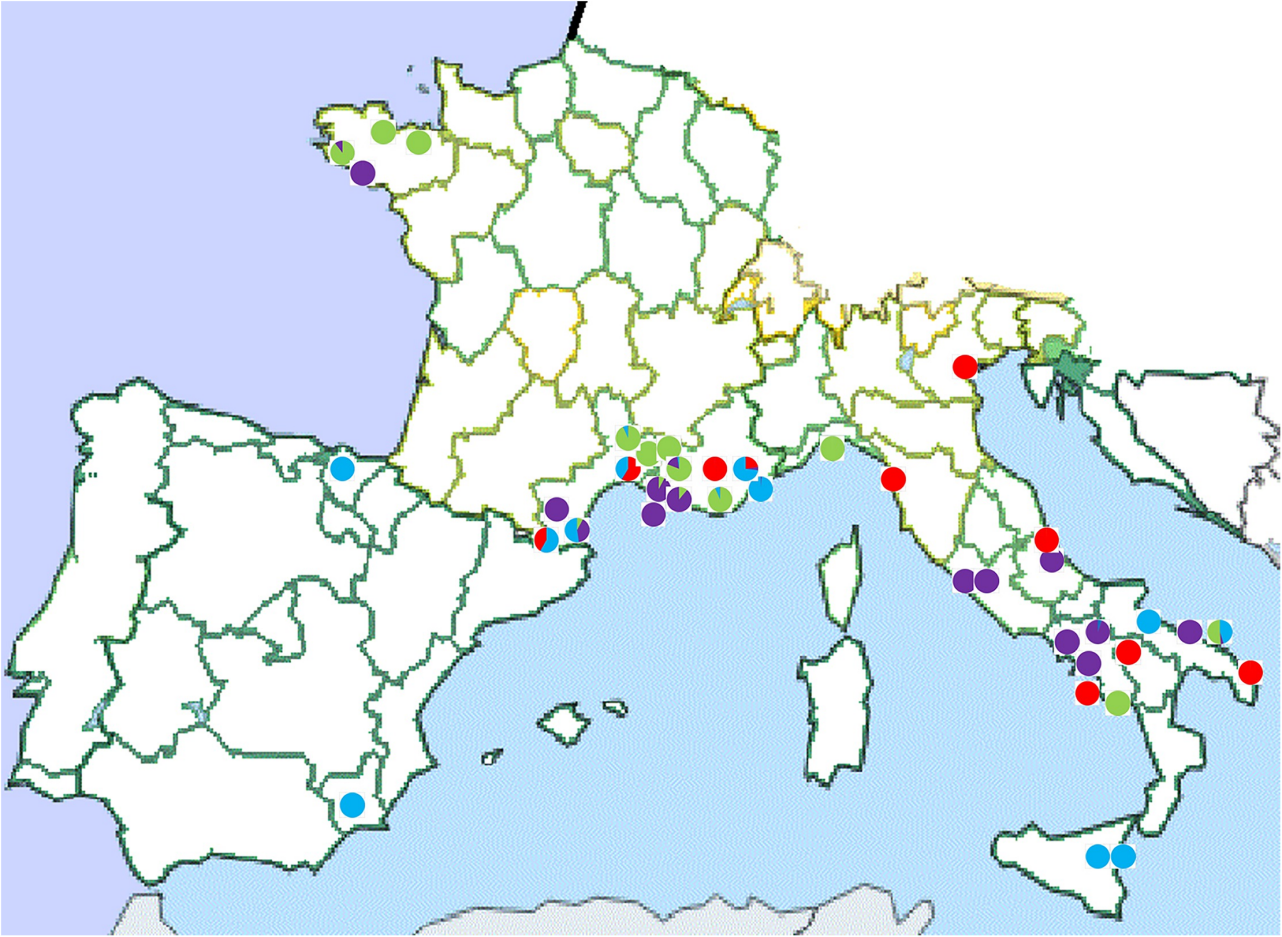
Map of the geographical distribution of the accessions well localized, showing the average proportion of the Q values inferred by STRUCTURE.

Geographical localization of some accessions was provided by the gene banks, which collected them. For the accessions geographically localized with certainty, the average proportions of the Q values obtained by STRUCTURE analysis were positioned on the geographical map (Fig 3). Even when a distribution pattern could be identified, such as the blue mainly in central Italy (Tyrrhenian side), the red mainly in Italy, the green in France, and turquoise in the southern part of each country, several exceptions were evident. It is interesting to note that the accessions from southern France have a multiple classification, belonging to more than a single group in most cases.

**Fig 4:**
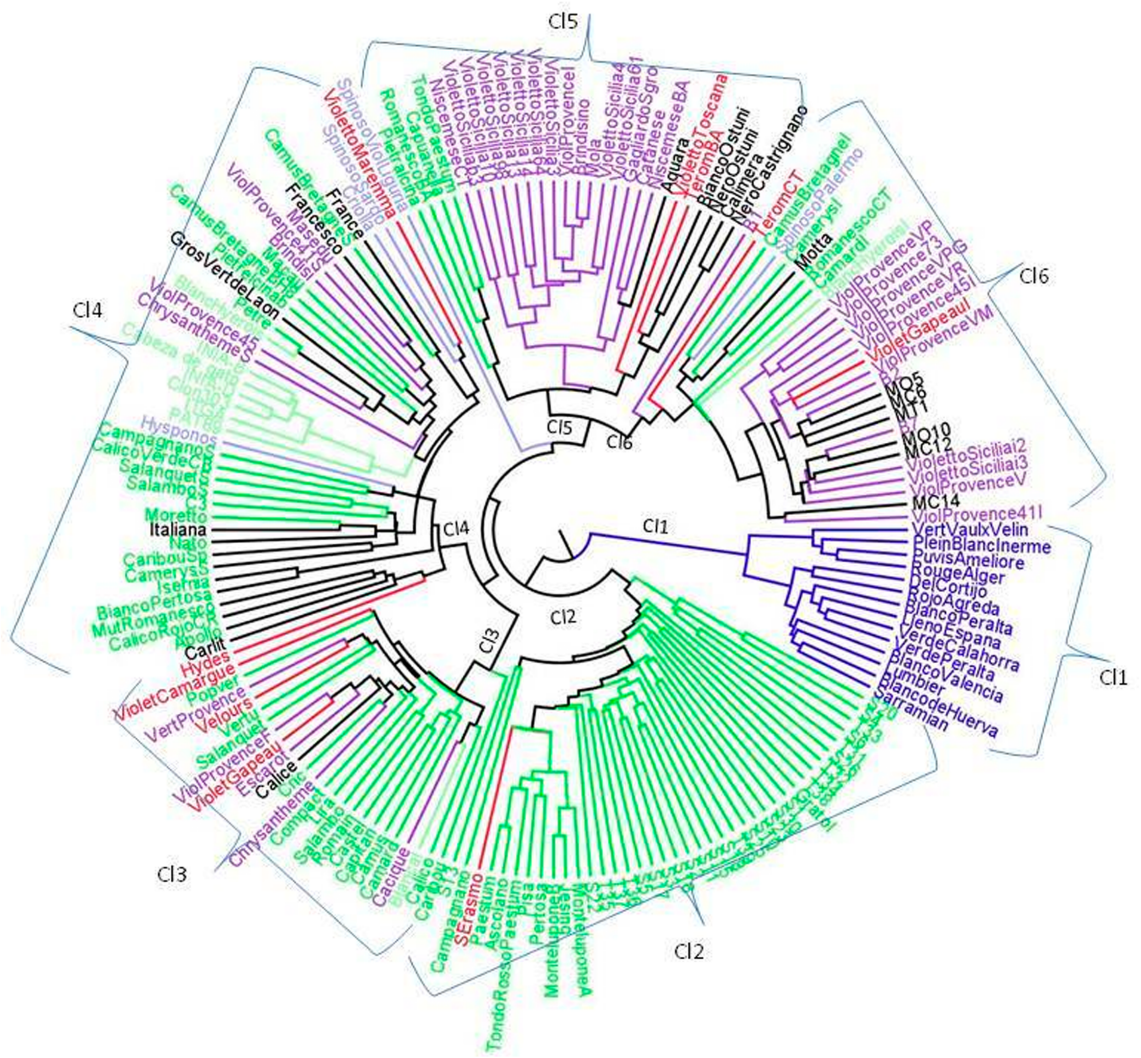
Cluster analyses of the 74 accessions using Nei’s [52] genetic distance and UPGMA. Different colors correspond to different accession typologies (see S1 Table for abbreviations and typologies).

Individual distance was computed using Nei genetic distance [52] or Euclidean distance based on all the data (dominant and co-dominant). The triangular matrices were then used for clustering analysis based on the UPGMA method using Power Markers (Fig 4). The clustering results were not very different for the two distance methods used, and so only the results obtained using Nei distance were presented. The dendrogram grouped the accessions into six clusters. The first group was CL1, which contained all the leafy cardoon accessions which, as expected, were well separated from all the others. The other five clusters included the globe artichoke accessions, which were divided mainly on the base of the collection. The CL2 group included accessions from the University of Viterbo collection (maintained at ARSIAL), which were mainly Romanesco accessions (36 out of 81), except for the S. Erasmo accession. CL3 contained most of the accessions from the French collection, including all the Violet and the Romanesco accessions, except Petre; while the other accessions from the French collection were close together in CL4 with all the Spanish accessions. CL5 and CL6 groups were close to each other and included all the accessions from CNR collections, which were mainly Violetto di Sicilia and Violet de Provence accessions, respectively, with a small group of Romanesco types and another of OFF types stemming from the CL5 group. The CL6 group included some Romanesco and one Spiny type.

**Fig 5:**
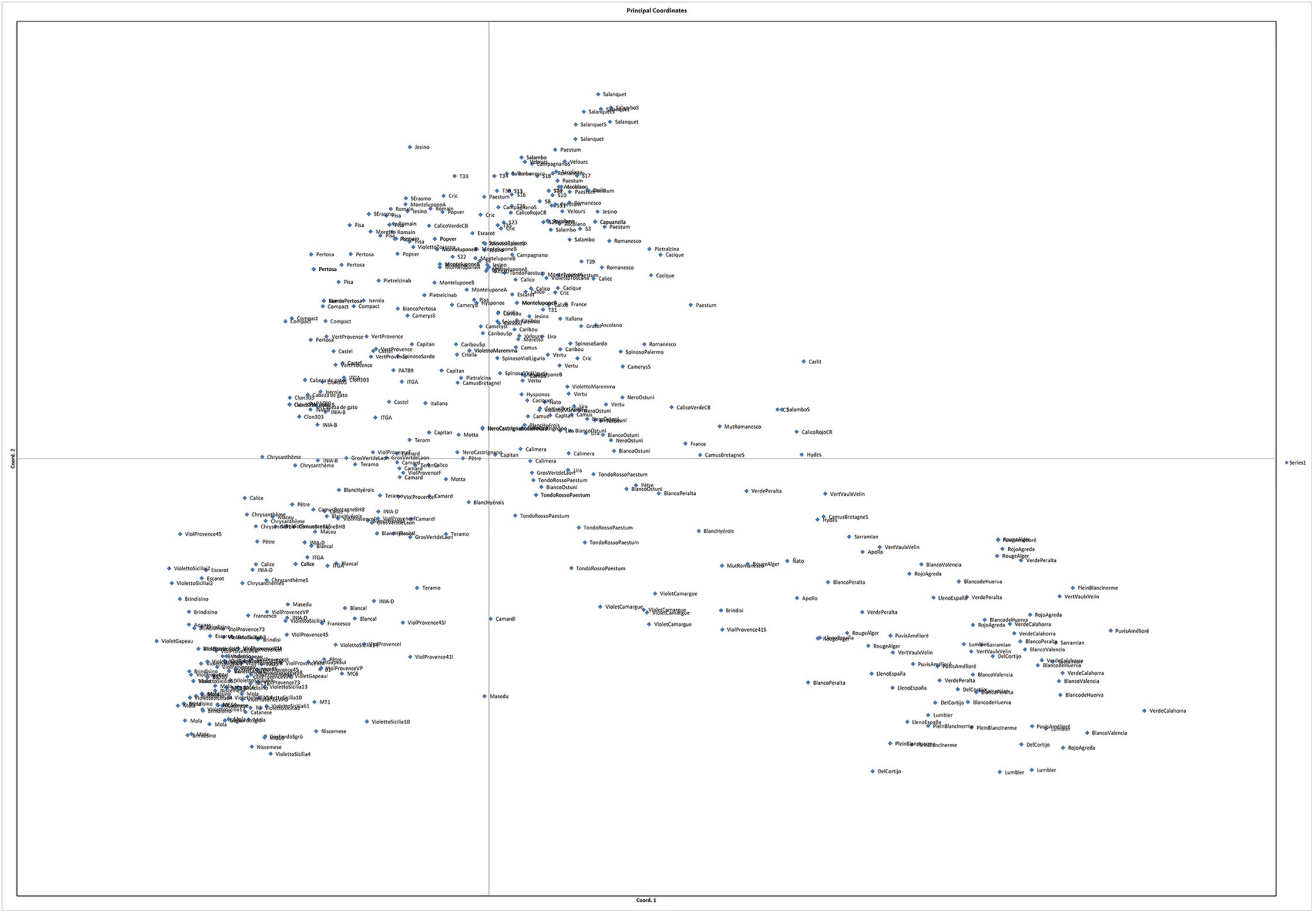
PCA via Covariance matrix with data standardization of co-dominant data.

Principal Coordinates Analysis (PCA) via Covariance matrix, based on co-dominant markers only, distributed the 556 individuals into a two-dimensional scatter plot. The first two PCA axes accounted for 32.39 and 23.60% of the genetic variation among accessions, respectively (Fig 5). Also in this case, the leafy cardoons were separate from the globe artichokes and were in the second quadrant of the graph with a positive PC1 and negative PC2. In the bottom left, with negative PC1 and PC2, were the Violet and Catanese types together with Tudela, which lay more towards the center. The Romanesco accessions were in the top central part of the plot (Fig 5). A similar distribution of individuals between the 2 PCA axes, accounting for about 48% of the genetic variation among accessions, was obtained using only dominant markers (S2 Fig), but in this case the Romanesco accessions were more spread along the PC1 axis.

### Genetic variance analysis

The hierarchical distribution of co-dominant molecular variance was partitioned among three levels: typology, accessions, and individuals. For typologies, seven groups, including the unassigned one, were used. The analysis revealed that only 15.49% of the total variation was among typologies, 19.22% was among accessions, but the greatest part, 65.29%, was within accessions (Table 3). Calculation of Wright’s *F* statistic at all SSR loci revealed that the genetic variation among accessions within the same geographical group (*F*_*SC*_) was 0.227, while among geographical groups (*F*_*CT*_) it was 0.155, and among accessions across the entire study area (*F*_*ST*_) it was 0.347. All the values were highly significant even if most of the variation was not among typologies.

**Table 3:** Analysis of MOlecular VAriance (AMOVA) considering individuals, accessions and typologies.

Source of variation	Degree of freedom	Sum of squares	Variance components	Percentage of variation
Among typologies	6	482.562	0.52542	15.49
Among accessions within typologies	167	1055.522	0.65182	19.22
Within accessions	938	2077.447	2.21476	65.29
Total	1111	3615.531	3.39200	

## Discussion

An assessment of genetic diversity is essential for understanding which germplasm should be conserved and/or what is being lost or could be in danger of extinction. The genetic diversity indicates how to build up a core collection, maximizing the variation among accessions and minimizing accession repetition. This is particularly important considering that cultivation strategies tend to concentrate on few varieties, with a consequent reduction in the cultivation of many local landraces. Moreover, a knowledge of population structure in a collection is a prerequisite for making association analyses to attribute allelic variation to important agronomic traits. Molecular markers may or may not correlate with the phenotypic expression of a genomic trait, but, whatever the case, they offer several advantages over conventional, phenotype-based alternatives. In particular, they are stable regardless of cultivation practices, environment, phenological phases or tissue. A structured collection analyzed with molecular markers is the basis for association studies involving phenotypic variation and biological function.

The germplasm analyzed here mainly derives from its center of origin [7], so it is not surprising that a huge diversity was detected in terms of polymorphic bands, expected and observed heterozyosity, fixation index, etc. The globe artichoke is mainly propagated vegetatively, and its outcrossing status is confirmed by the high level of heterozygosity observed which is on average slightly less than 50%. In the present study, the use of sequencer machines to read markers enabled us to discriminate the bands with precision, while some of the previous studies only used silver staining and visual recording. The sequencers also made it easier to rerun the analysis to confirm the recording; moreover, the results from different laboratories were compared to validate the data used.

### Markers and identified diversities

Among the markers used, some are more informative than others, as indicated by the different parameters used. The SSR CLIB-02 should certainly be included in any *C*. *cardunculus* evaluation since it was one of the most variable markers (Table 1); also good markers, in increasing order of PIC, were: CsPal02, CMAL-108, CMAL21, CMAFLP-04, CMAFLP-18, CMAL06, CsPal03, CLIB-12, CMAL24, and CDAT-01. By contrast, CMAFLP-05 did not provide useful information. It should be pointed out that CMAL-25, even with a PIC value below the average, was not in LD with any of the others. The PIC value of the dominant markers was much lower than that of the co-dominant markers, ranging from 0.176 for EagcMctt to 0.242 for EacgMctt (average 0.211) in the case of AFLP and from 0.181 for 834 to 0.277 for 857c (average 0.235) in the case of ISSR (Table 1). The present results showed that the differences within the two marker typologies (ISSR and AFLP) were much more consistent than those found by Lanteri et al. [29] and are similar to those found by Pagnotta et al. [30].

The high level of heterozygosity detected was expected since *C*. *cardunculus* is a highly outcrossed species. The level of He and Ho detected here (Table 1) in the SSR of the “CMAL-” and “CsPal-” series are comparable with the values found by Acquadro et al. [47] and Sonnante et al. [39], who tested newly developed SSR markers on a core collection of 27 or 29 globe artichoke accessions, respectively, but also on leafy cardoon accessions. The values of He and Ho and the number of alleles found here were much higher than the corresponding values found by Acquadro et al. [48], who tested their newly identified SSR of “CDAT-” and CLIB-” series on a slightly smaller core collection, as well as the value for CsCiCaca05 detected by Sonnante et al. [39]. Conversely, for the SSR of the “CMAFLP-” group, the values of the above parameters was much lower than those detected by Acquadro et al. [40], who mainly tested wild cardoon (24 accessions), and a limited number of leafy cardoon and globe artichoke (2 accessions each). Despite the high number of amplicons detected by the dominant markers (S7 Table), and hence the possibility to explore a wider genomic region, they were less informative (in terms of PIC) than SSRs due to their uncertain allelic phase [23]. Moreover, the dominant markers were considered to have only two possible alleles, i.e. band presence or absence. In this case, the He detected indicates that one of the two allele is quite common (with a frequency of about 80%) and the other rarer (frequency of about 20%). In fact, an He equal to 0.257 (average level for AFLP) means that the two alleles have a frequency of about 0.152 and 0.848 respectively. Similarly, in the case of ISSR, the average He was equal to 0.284 (Table 1) indicating that the allele frequencies were equal to about 0.171 and 0.829 for the two possible alleles.

It is interesting to note that a high number of alleles were identified for the co-dominant markers, with an average of eight alleles (excluding the monomorphic) per marker and a maximum of 15 alleles per marker (Table 1). An average of 18.3 alleles per locus was found by Gatto et al. [8], even though their study also included wild cardoon besides globe artichoke and leafy cardoon, while about six alleles were detected by Portis et al. [60] in wild cardoon and by Scaglione et al. [13] in a core collection with all the three *C*. *cardunculus* taxa. A much lower number was found by Crinò and Pagnotta [61] analyzing a collection including only Romanesco artichoke accessions from Latium region (Italy). Conversely, the number of alleles found in our work was similar to that detected by Ben Ammar et al. [19] in wild cardoon. This was also true when the globe artichoke and the leafy cardoon accessions were considered separately, even if, in these cases, the values were lower (S3 and S4 Tables).

### Accessions and identified diversities

It is important to point out that despite the many alleles identified in the entire germplasm (Table 1), very few alleles were found in each accession (S7 Table), i.e. the alleles common to many accessions are limited. In addition, 34 alleles (Table 2) were specific for a single accession (private allele), which might be particularly useful for discriminating and fingerprinting the relative accession.

The average values for the *F*_*IS*_, *F*_*IT*_, and *F*_*ST*_ were -0.66, 0.16, and 0.51, respectively (Table 1). The variance of allele frequencies among accessions (*F*_*ST*_) detected here is higher than the average values found in wild cardoon [19, 60] or in *C*. *cardunculus* [8]. This is true also if the globe artichoke and the leafy cardoon accessions were considered separately, even if in these cases the values were lower (S3 and S4 Tables). The fixation indexes across accessions were mainly negative, ranging from -1 to 0.24 with an average of -0.74. Since the fixation index is equal to (H_e_-H_o_) ÷ H_e_ = 1-(H_o_ ÷ H_e_), its values ranged from -1 to +1. Values close to zero are expected with random mating, while substantial positive values indicate inbreeding or undetected null alleles. Negative values, as were here found, indicate an excess of heterozygosity due to negative assortative mating or selection for heterozygotes, which is not surprising in a highly outcrossing species like *C*. *cardunculus*. This is particularly true for globe artichoke (overall average F = -0.798), while for leafy cardoon (overall average F = -0.032) the values were among the highest, indicating random mating. This difference can be explained with the different propagation system, clonal for globe artichoke, by seed for leafy cardoon as for wild cardoon [12].

It is interesting to note that C3 was the accession with the lower level of observed heterozygosity. C3 clone has come to be widely cultivated in Italy in the last 10 years since micro-propagation was started, enabling its multiplication at a high rate [61]. It should be considered that a variable number of individuals per accession were analyzed, and this could have affected the computed parameters, particularly for the accessions with a single individual, such as C3. Nevertheless, it should be pointed out that correlation analyses between the number of individuals and the different computed parameters (data not shown) did not highlight any significance.

### Relationships among accessions

All the previously published works analyzed *C*. *cardunculus* accessions belonging to single collections. Not surprisingly, they generally revealed good discrimination among the three *C*. *cardunculus* taxa, separating the globe artichoke from the leafy cardoon and/or the wild cardoon [8, 13, 32, 34, 37–40, 43]. In this study, using a higher number of accessions, a similar result was obtained comparing globe artichoke *versus* leafy cardoon accessions. The globe artichoke and the leafy cardoon displayed morphological and genetic differences, so it is surprising that, according to UPOV, the two taxa are bulked in the same description protocols. Moreover, the two taxa are differentiated by their reproductive habits [9] and for the commercial parts used, i.e. the leaves for the leafy cardoon and the capitula for the globe artichoke.

In a previous study, the STRUCTURE analysis in *C*. *cardunculus* was run only in a collection that mainly included wild cardoon (43 accessions) and a limited number of leafy cardoon (10 accessions) and globe artichoke (16 accessions) [8]. In the present study, the leafy cardoon accessions were separated into one sub-group and the globe artichoke accessions were differentiated in five sub-groups according to their structure. In this case, as in Gatto et al. [8], the Romanesco types were heterogeneously grouped, with the Spiny and Violet types not well distinguishable from some of the Romanesco. Catanese types are in a sub-group close to the Tudela type. Also, as in Gatto et al. [8], only the Catanese groups of the globe artichoke accessions showed consistency. Not surprisingly, the unassigned individuals were spread among four of the five globe artichoke sub-groups. Generally, all the individuals of a single accession were grouped together. Nevertheless, there are some exceptions in which individuals belonging to the same accession are assigned differently. These were the two Brindisi accessions, one blue and the other turquoise; and the Escarot, Pètre, Capitan, Compact, Hydes and MutRomanesco accessions, which shared more colors but inconsistently among individuals (Fig 2).

The resulting geographical distributions obtained by mapping the Q values, only for the globe artichoke accessions with a defined cultivation area, showed no clear pattern. Even if in some geographic areas some colors seemed to predominate (Fig 3). Most of the accessions from southern France have a multiple classification and belonged to more than one group, indicating the low uniformity of these accessions.

The dendrogram obtained with the Nei [52] genetic distance (Fig 4) grouped the accessions into 6 clusters, but, surprisingly, except for the leafy cardoon group, there was no consistency in the accession typologies, unlike that mentioned in other publications. Conversely, the origin of the collections seems to be more important. The CL3 and CL4 groups mainly included the accessions from France and Spain, respectively. The CL2 group included mainly Romanesco accessions, which were mainly from Central Italy gene banks. CL5 and CL6 included both Catanese and Violet types, which come from Southern Italy gene banks.

Within the groups, the leafy cardoon accessions (CL1) were clearly sub-grouped into leafy cardoon from France and leafy cardoon from Spain, in agreement with previous works, in which leafy cardoon accessions were placed in distinct groups according to their geographical origin [8, 13, 37, 62]. As for the globe artichoke samples, the French accessions in CL3 were roughly divided into the Romanesco and Violet types. The Spanish accessions in CL4 were clearly subdivided into Tudela and Romanesco types. To the best of our knowledge, this is the first time that a wide collection of Tudela accessions has been analyzed and it is not surprising to find them well distinguished from the others. Previous studies pointed to the admixture structure of Blanca de Tudela lying between Catanese and Romanesco types [8], or allocated it close to Locale di Sibari [42]. The Spanish accessions of the Catanese and Spiny typologies were not markedly divided in CL4 either. Regarding the groups CL5 and CL6, as far as we know, this is the first time that several Violet de Provence accessions have been analyzed, except in Sonnante et al. [39] and Gatto et al. [8], where a single accession from Italy was included and where, in the first case, no clustering was reported. The overlapping of Spiny with Romanesco and the Violet with Catanese was also found by Sonnante et al. [38], while Scaglione et al. [13], using an EST database, grouped the Violet and Spiny types separately on one side and the non-Spiny types on the other.

The Principal component analysis (Fig 5) dealing with only two main coordinates, which accounted both for about half of the total variation, clearly separated the leafy cardoon accessions, while all the other 484 individuals were spread along the two axes, with no clear pattern. This is also because, as underlined by the AMOVA analysis (with co-dominant markers), most of the variation was within the accessions and only 15.5% was due to differences among typologies. This was in line with previous results provided by Lanteri et al. [24], who, in Spinoso Sardo accessions, detected that 72% of the variation was due to within-accessions variations. Conversely, De Felice et al. [42] attributed most of the variation (86%) among accessions, and in Raccuia et al. [32] the results depended mainly on the groups analyzed. The percentage of variation among and within accessions was strongly related to the species breeding system, outcrossing species like *C*. *cardunculus* showing a higher variation within populations (accession) (see Pagnotta et al. [63] and references therein).

## Conclusion

This is the first study that jointly analyzes *C*. *cardunculus* germplasm from different European regions and collections. Due to the high degree of heterozygosity and the vegetative propagation system of the globe artichoke, the structure of the accessions was only partially in agreement with some important morphological traits, which are the basis for the classification of accessions into typologies. In general, the grouping results were mainly typology-based. However, in spite of the great diversity found in the collections, it seems that the method adopted to conserve the germplasm affected their genetic distance and, indeed, the origin of the collections was reflected in germplasm clustering. It should be said that the origin often coincides with the main typologies. Hence, the assessment of genetic diversity could not be unequivocally detected when studies dealt with few accessions or accessions coming from the same collection.

The present results highlight that particular attention should be paid to the method used to conserve the *C*. *cardunculus* germplasm. The globe artichoke is mainly propagated vegetatively and the germplasm is conserved in field gene banks, which are subject to environmental effects. The different environmental conditions present in the field gene banks located in France, Spain, Central Italy and Southern Italy may well have created different selective pressures, which fix common alleles regardless of the accession type. Strategies to mitigate the environmental effects on field gene banks should apply several conservation methods including *in vitro* and cryopreservation, which are less affected by environmental conditions. As a conclusion, the accessions sharing the same or similar name should not be considered similar by default but great care should be taken to identify their origin and conservation methodology.

In the future, the present wide collection, which is structured according to genetic analyses, could serve as a base for association studies knowing its morphological characterization as well. Moreover, to run association maps and to pick higher diversity among accessions it is advisable to include accessions from different gene banks in the corresponding analysis.

## Supporting information

S1 Fig Optimal value of K determined by the magnitude of delta K (ΔK) as a function of K (mean ± SD over 20 replicates). (TIF)

S2 Fig PCA via Covariance matrix with data standardization of dominant data. (TIF)

S1 Table List of the *Cynara cardunculus* germplasm ordered by typology and abbreviation name. (DOCX)

S2 Table ISSR primers and their annealing temperatures (T). (DOCX)

S3 Table Markers used to genotype 484 *Cynara cardunculus* var. *scolymus* individuals with their genetic diversity parameters. Number of bands or alleles in the case of SSR, frequency of the principal allele (MAF), Gene diversity and Expected Heterozygosity.
(DOCX)

S4 Table Markers used to genotype 72 *Cynara cardunculus* var. *altilis* individuals with their genetic diversity parameters. Number of bands or alleles in the case of SSR, frequency of the principal allele (MAF), Gene diversity and Expected Heterozygosity.
(DOCX)

S5 Table Significant linkage disequilibrium (significance level = 0.0500) between loci indicated by +. (DOCX)

S6 Table Accession diversity measured with dominant (D) or co-dominant (CD) markers. Polymorphism (P), expected heterozygosity (He), observed heterozygosity (Ho), Fixation Index (F), Number of alleles with frequency higher than 5% (Na), Shannon's Information Index (I), and number of less common Alleles (< = 25%) (No).
(DOCX)

S7 Table Average value per accession for each markers used to genotype *Cynara cardunculus* individuals. Number of alleles (Na), Number of effective alleles (Ne), Shannon's Information Index (I), expected heterozygosity (He), Unbiased Expected Heterozygosity (UHe), and amplicons in the case of dominant markers.
(DOCX)

## References

[1] CPVO-TP 184/2 Globe artichoke [Internet]. 2013. http://www.cpvo.europa.eu/documents/TP/veg/TP_CYNARA_CARDUNCULUS_184-2.pdf

[2] Porceddu E, Della Cecca V, Bianco VV. Classificazione numerica di cultivar di carciofo. In: Proceedings II International Congress of Artichoke. Bari (Italy). Minerva Medica (ed.), Turin, 1976; p. 1105–1119.

[3] Cravero V, Martin E, Lopez Anido F, Cointry E. Stability through years in a non-balanced trial of globe artichoke varietal types. Sci Hort. 2010;126: 73–79.

[4] FAOSTAT. 2016. Production 2014 (Update). [Internet]. http://faostat3.fao.org/browse/Q/QC/E

[5] Pagnotta MA, Noorani A. Genetic Diversity Assessment in European Cynara Collections. In Tuberosa R, Graner A, Frison E. Editors. Genomics of Plant Genetic Resources. 2014; P. 559–584. ISBN: 978-94-007-7571-8 (Print) 978-94-007-7572-5 (Online)

[6] Foury C. Ressources genetiques et diversification de l’artichaut (*Cynara scolymus* L.). Acta Hort. 1989;242: 155–166.

[7] Pignone D, Sonnante G. Wild artichokes of south Italy: did the story begin here? Genet Resour Crop Ev. 2004;51: 577–580.

[8] Gatto A, De Paola D, Bagnoli F, Vendramin GG, Sonnante G. Population structure of *Cynara cardunculus* complex and the origin of the conspecific crops artichoke and cardoon. Ann Bot. 2013;112(5): 855–865. doi: 10.1093/aob/mct150 23877076 10.1093/aob/mct150 PMC3747803

[9] Sonnante G, Pignone D, Hammer K. The domestication of artichoke and cardoon: from Roman times to the genomic age. Ann Bot. 2007;100(5): 1095–1100. doi: 10.1093/aob/mcm127 17611191 10.1093/aob/mcm127 PMC2759203

[10] Pandino G, Lombardo S, Mauro RP, Mauromicale G. Variation in polyphenol profile and head morphology among clones of globe artichoke selected from a landrace. Sci. Hortic. 2012;138: 259–265.

[11] Snyder MJ. Investigation of propagational techniques for artichoke. In: Atti 3nd Congr Int Stud Carciof, Bari Ind Grafica Laterza, Bari. 1979; p. 347–358.

[12] Sonnante G, Gatto A, Morgese A, Montemurro F, Sarli G, Blanco E, et al Genetic map of artichoke × wild cardoon: toward a consensus map for *Cynara cardunculus*. Theor Appl Genet. 2011;123: 1215–1229. doi: 10.1007/s00122-011-1661-1 21800142 10.1007/s00122-011-1661-1

[13] Scaglione D, Acquadro A, Portis E, Taylor CA, Lanteri S, Knapp SJ. Ontology and diversity of transcript-associated microsatellites mined from a globe artichoke EST database. BMC Genomics. 2009;10(1): 1. 19785740 10.1186/1471-2164-10-454 PMC2760586

[14] Bianco VV. Carciofo (Cynara scolymus L.). In: Bianco VV, Pampini F editors. Orticoltura, Patron Editore, Bologna; 1990. P. 209–251. Italian.

[15] Crinò P, Tavazza R, Rey Muñoz NA, Trionfetti Nisini P, Saccardo F, Ancora G et al Recovery, morphological and molecular characterization of globe artichoke ‘Romanesco’ landraces. Genet Resour Crop Ev. 2008;55 (6): 823–833.

[16] Noorani A, Rey N, Temperini A, Temperini O, Dulloo E, Saccardo F, et al Assessment of genetic variation in three populations of Italian wild cardoon. Acta Hort. 2012;942: 49–54.

[17] Noorani A, Rey N, Mondini L, Ciancolini A, Crinò P, Pagnotta MA. Morphological diversity assessment in wild and cultivated cardoons. Acta Hort. 2013;983, 47–54.

[18] Boury S, Jacob A-ME, Egea-Gilabert C, Fernández JA, Sonnante G, Pignone D. et al Assessment of Genetic Variation in an Artichoke European Collection by Means of Molecular Markers. Acta Hort. 2012;942: 81–87.

[19] Ben Ammar I, Sonnante G, Dridi BAM. Genetic variability in wild cardoon (*Cynara cardunculus* L. var. *sylvestris*) revealed by SSR markers and morphological traits. Sci Hortic. 2015;185, 76–81.

[20] Frankel OH. Genetic prospectives of germplasm conservation In: Arber WK editor. Genetic manipulation: impact on man and society. Cambridge University Press, Cambridge 1984; p. 161–170.

[21] Brown AHD. The case for core collections In: Brown AHD, Frankel DH, Marshall DR, Williams JT editors. The use of plant genetic resources. Cambridge University Press, Cambridge 1989a; P. 136–156.

[22] Brown AHD. Core collections: a practical approach to genetic resources management. Genome. 1989b;31: 818–824.

[23] Mondini L, Noorani A, Pagnotta MA. Assessing Plant Genetic Diversity by Molecular Tools. Diversity. 2009;1: 19–35.

[24] Lanteri S, Di Leo I, Ledda L, Mameli MG, Portis E. RAPD variation within and among populations of globe artichoke cultivar ‘Spinoso sardo’. Plant Breed. 2001;120: 243–246.

[25] Sonnante G, De Paolis A, Lattanzio V, Perrino P. Genetic variation in wild and cultivated artichoke revealed by RAPD markers. Genet Res Crop Ev. 2002;49: 247–252.

[26] Messmer M, Scheider E, Stekly G, Büter B. Determination of the Progenitors and the Genetic Stability of the Artichoke Cultivar SALUSCHOCKE® Using Molecular Markers. Journal of Herbs, Spices and Medicinal Plants. 2002;9(2–3): 177–182.

[27] Tivang J, Skroch P, Nienhuis J, De Vos N. Randomly amplified polymorphic DNA (RAPD) variation among and within artichoke (*Cynara scolymus* L.) cultivars and breeding populations. J Amer Hort Sci. 1996;121: 783–788.

[28] Sonnante G, Ippedico M, De Paolis A. Microsatellite and AFLP markers in an artichoke world collection. Acta Hort. 2004b;660: 61–68.

[29] Lanteri S, Acquadro A, Saba E, Portis E. Molecular fingerprinting and evaluation of genetic distances among selected clones of globe artichoke (*Cynara cardunculus* L. var. *scolymus* L.). J Hortic Sci Biotech. 2004;79: 863–870.

[30] Pagnotta MA, Cardarelli MT, Rey NA, Tucci M, Saccardo F. Assessment of genetic variation in artichoke of ‘Romanesco’ type by molecular markers. Acta Hort. 2004;660: 99–104.

[31] Ciancolini A, Rey NA, Pagnotta MA, Crinò P. Characterization of Italian spring globe artichoke germplasm: morphological and molecular profiles. Euphytica. 2012;186(2): 433–443.

[32] Raccuia SA, Mainolfi A, Mandolino G, Melilli MG. Genetic diversity in *Cynara cardunculus* revealed by AFLP markers: comparison between cultivars and wild types from Sicily. Plant Breed. 2004;123: 280–284.

[33] Portis E, Mauromicale G, Barchi L, Mauro R, Lanteri S. Population structure and genetic variation in autochthonous globe artichoke germplasm from Sicily Island. Plant Sci. 2005;168(6): 1591–1598.

[34] Mauro R, Portis E, Acquadro A, Lombardo S, Mauromicale G, Lanteri S. Genetic diversity of globe artichoke landraces from Sicilian small-holdings: implications for evolution and domestication of the species. Conserv Genet. 2009;10(2): 431–440.

[35] Acquadro A, Papanice MA, Lanteri S, Bottalico G, Portis E, Campanale A, et al Production and fingerprinting of virus-free clones in a reflowering globe artichoke. Plant Cell Tissue Organ Cult. 2010;100: 329–337.

[36] Lo Bianco CL, Fernández JA, Migliaro D, Crinò P, Egea-Gilabert C. Identification of F1 hybrids of artichoke by ISSR markers and morphological analysis. Mol Breed. 2011;27(2): 157–170.

[37] Lahoz I, Fernández JA, Migliaro D, Macua JI, Egea-Gilabert C. Using molecular markers, nutritional traits and field performance data to characterize cultivated cardoon germplasm resources. Sci Hortic. 2011;127(3): 188–197.

[38] Sonnante G, De Paolis A, Pignone D. Relationships among artichoke cultivars and some related wild taxa based on AFLP markers. Plant Genet Resour Charact Util. 2004;1: 125–133.

[39] Sonnante G, Carluccio AV, De Paolis A, Pignone D. Identification of artichoke SSR markers: molecular variation and patterns of diversity in genetically cohesive taxa and wild allies. Genet Resour Crop Ev. 2008;55: 1029–1046.

[40] Acquadro A, Portis E, Albertini E, Lanteri S. M-AFLP-based protocol for microsatellite loci isolation in *Cynara cardunculus* L. (Asteraceae). Mol Ecol Notes 2005;5: 272–274.

[41] Curci PL, De Paola D, Sonnante G. Development of chloroplast genomic resources for Cynara. Mol Ecol Resour. 2015;16 (2): 562–573. doi: 10.1111/1755-0998.12457 26354522 10.1111/1755-0998.12457

[42] De Felice B, Borra M, Manfellotto F, Anna S, Biffali E, Guida M. Assessment of genetic diversity between wild and cultivated artichokes using SSR markers. Genet Resour Crop Ev. 2015; 1–7

[43] Cravero V, Martín E, Cointry E. Genetic diversity in *Cynara cardunculus* determined by sequence-related amplified polymorphism markers. J Amer Soc Hort Sci. 2007;132(2): 208–212.

[44] Hedrick PW. Gametic disequilibrium measures: proceed with caution. Genetics. 1987;117(2): 331–341. 3666445 10.1093/genetics/117.2.331 PMC1203208

[45] Pritchard JK, Stevens M, Donnelly P. Inference of population structure using multi-locus genotype data. Genetics. 2000;155: 945–959. 10835412 10.1093/genetics/155.2.945 PMC1461096

[46] Vos P, Hogers R, Bleeker M, Reijans M, Van de Lee T, Homes M, et al AFLP: a new technique for DNA fingerprinting. Nucleic Acids Res. 1995;23(21): 4407–4414. 7501463 10.1093/nar/23.21.4407 PMC307397

[47] Acquadro A, Portis E, Lee D, Donini P, Lanteri S. Development and characterization of microsatellite markers in *Cynara cardunculus* L. Genome. 2005b;48(2): 217–225. 15838543 10.1139/g04-111

[48] Acquadro A, Portis E, Lanteri S. Isolation of microsatellite loci in artichoke (*Cynara cardunculus* L. var. *scolymus*). Mol Ecol Notes. 2003;3(1): 37–39.

[49] Nei M. Analysis of gene diversity in subdivided populations. Proc Nat Acad Sci USA. 1973;70: 3321–3323. 4519626 10.1073/pnas.70.12.3321 PMC427228

[50] Guo X, Elston RC. Linkage informative content of polymorphic genetic markers. Hum Hered. 1999;49: 112–118. doi: 22855 10077733 10.1159/000022855

[51] Wright SW. The interpretation of population structure by F-statistics with special regard to systems of mating. Evolution. 1965;19: 358–420.

[52] Nei M. Genetic Distance between Populations. The American Naturalist. 1972;106: 283–292.

[53] Engelman L, Hartigan JA. K-Means Clustering In Dixon WJ, Engelman L, Frane JW, Hill MA, Jennrich RI, Toporek JO editors. BMDP Statistical software Manual. University of California Press, Berkely USA 1985; p. 464–478.

[54] Evanno G, Regnaut S, Goudet J. Detecting the number of clusters of individuals using the software STRUCTURE: a simulation study. Mol Ecol. 2005;14: 2611–2620. doi: 10.1111/j.1365-294X.2005.02553.x 15969739 10.1111/j.1365-294X.2005.02553.x

[55] Lewontin RC, Kojima K. The evolutionary dynamics of complex polymorphisms. Evolution. 1960;14: 458–72.

[56] Peakall R, Smouse PE. GENALEX 6: genetic analysis in Excel. Population genetic software for teaching and research. Mol Ecol Notes. 2006;6: 288–295. 10.1093/bioinformatics/bts460 PMC3463245 22820204

[57] Peakall R. Smouse PE. GenAlEx 6.5: genetic analysis in Excel. Population genetic software for teaching and research—an update. Bioinformatics. 2012;28: 2537–2539. doi: 10.1093/bioinformatics/bts460 22820204 10.1093/bioinformatics/bts460 PMC3463245

[58] Liu K, Muse SV. PowerMarker: an integrated analysis environment for genetic marker analysis. Bioinformatics. 2005;21(9): 2128–9. doi: 10.1093/bioinformatics/bti282 15705655 10.1093/bioinformatics/bti282

[59] Excoffier L, Laval G, Schneider S. Arlequin ver. 3.0: An integrated software package for population genetics data analysis. Evolutionary Bioinformatics Online. 2005;1: 47–50. PMC2658868 19325852

[60] Portis E, Acquadro A, Comino C, Mauromicale G, Saba E, Lanteri S. Genetic structure of island populations of wild cardoon [*Cynara cardunculus* L. var. *sylvestris* (Lamk) Fiori] detected by AFLPs and SSRs. Plant Sci. 2005b;169(1): 199–210.

[61] Crinò P, Pagnotta MA. Phenotyping, Genotyping, and Selections within Italian Local Landraces of Romanesco Globe Artichoke. Diversity 2017; 9, 14: 1–15.

[62] Portis E, Barchi L, Acquadro A, Macua JI, Lanteri S. Genetic diversity assessment in cultivated cardoon by AFLP (Amplified Fragment Length Polymorphism) and microsatellite markers. Plant Breed. 2005c;124(3): 299–304.

[63] Pagnotta MA, Cocks PS, Snaydon RW. The breeding systems of three annual clovers native to north Syria. Israel J Plant Sci. 1995;43: 347–358.

