# Supplementary figures and images for: Genetic diversity and accession structure in European Cynara cardunculus collections
Source: PLoS One. 2017 Jun 1;12(6):e0178770. doi: 10.1371/journal.pone.0178770 (PMC5453587; doi:10.1371/journal.pone.0178770)

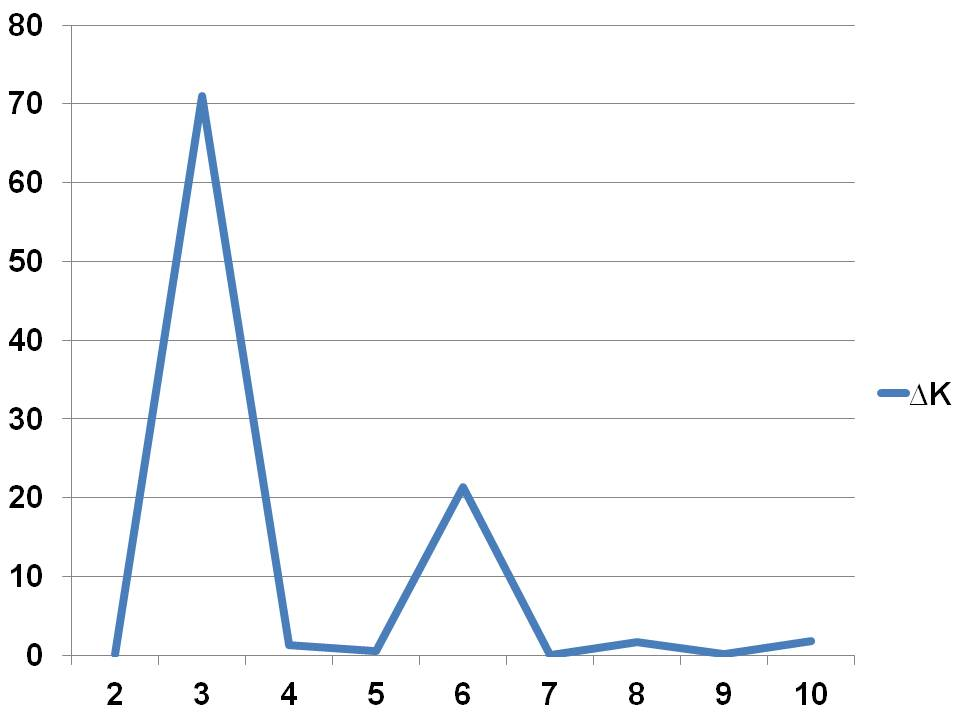

Supplement: S1 Fig — (TIF) [file pone.0178770.s001.tif]

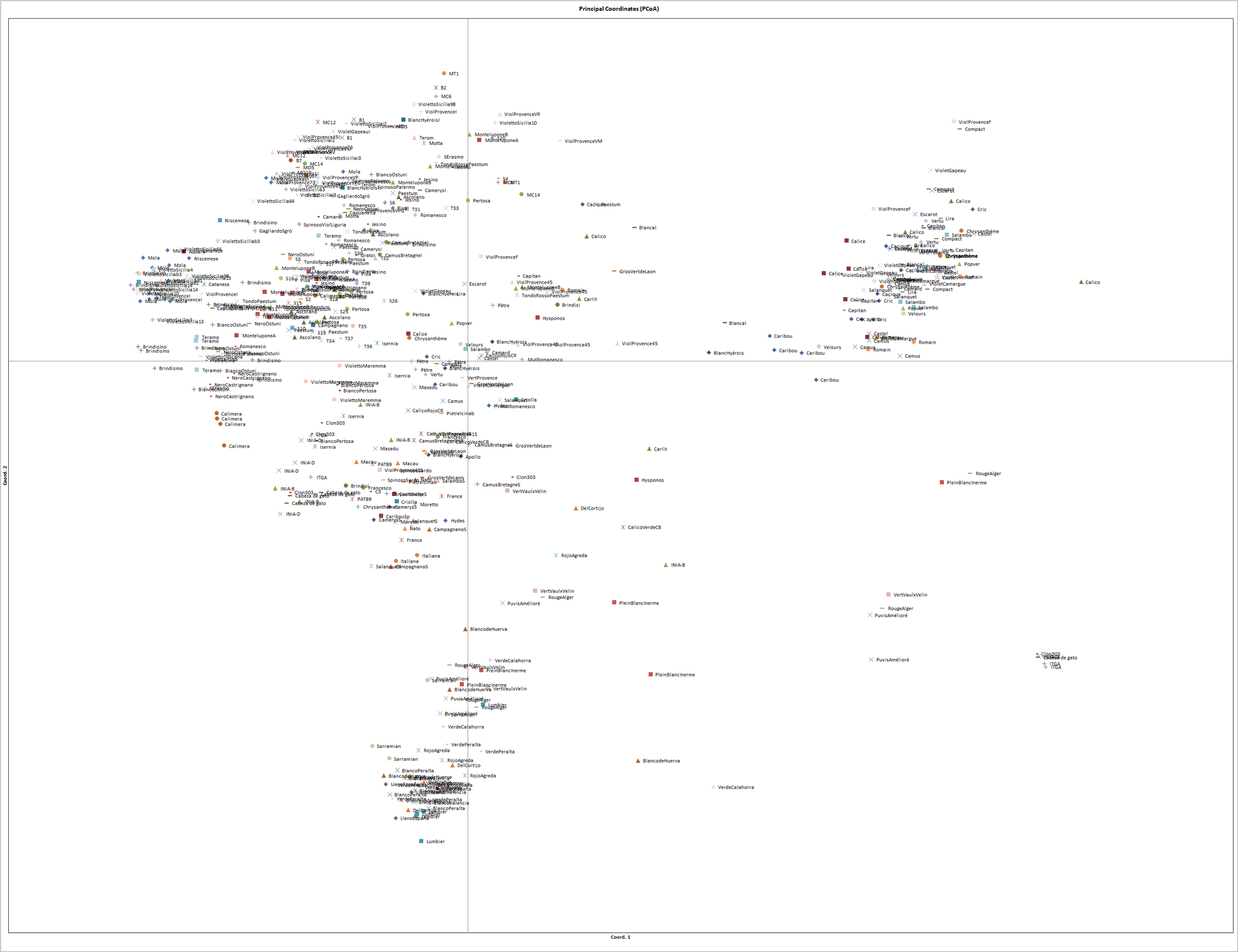

Supplement: S2 Fig — (TIF) [file pone.0178770.s002.tif]
