# Supplementary material for: Genetic diversity and accession structure in European Cynara cardunculus collections
Source: PLoS One. 2017 Jun 1;12(6):e0178770. doi: 10.1371/journal.pone.0178770 (PMC5453587; doi:10.1371/journal.pone.0178770)
Supplement: S1 Table — (DOCX) [file pone.0178770.s003.docx]

S1 Table. List of the *Cynara cardunculus* germplasm ordered by typology and abbreviation name.

| **Accession** | **Origin** | **ngenotypes** | **Abreviation** | | **type** |  |
| --- | --- | --- | --- | --- | --- | --- |
| Blanco de Huerva | Spain | 5 | BlancodeHuerva | | Cardoon |  |
| Blanco Peralta | Spain | 5 | BlancoPeralta | | Cardoon |  |
| Blanco Valencia | Spain | 5 | BlancoValencia | | Cardoon |  |
| Del cortijo | Spain | 5 | DelCortijo | | Cardoon |  |
| Lleno de España | Spain | 5 | LlenoEspaña | | Cardoon |  |
| Lumbier | Spain | 5 | Lumbier | | Cardoon |  |
| Plein Blanc inerme | France | 5 | PleinBlancInerme | | Cardoon |  |
| Puvis Amélioré | France | 6 | PuvisAmélioré | | Cardoon |  |
| Rojo de Agreda | Spain | 5 | RojoAgreda | | Cardoon |  |
| Rouge D'Alger bis | France | 6 | RougeAlger | | Cardoon |  |
| Sarramian | Spain | 5 | Sarramian | | Cardoon |  |
| Verde Calahorra | Spain | 5 | VerdeCalahorra | | Cardoon |  |
| Verde de Peralta | Spain | 5 | VerdePeralta | | Cardoon |  |
| Vert de Vaulx en Velin | France | 5 | VertVaulxVelin | | Cardoon |  |
| Brindisi | Spain | 2 | Brindisi | | Catanese |  |
| Brindisino PT | Bari - Italy | 14 | Brindisino | | Catanese |  |
| Cacique | France | 4 | Cacique | | Catanese |  |
| Catanese | Bari - Italy | 2 | Catanese | | Catanese |  |
| Chrysanthème | France | 5 | Chrysanthème | | Catanese |  |
| Crysantheme Sp | Spain | 2 | ChrysanthèmeS | | Catanese |  |
| CT- ViolProvence Vpmag | Bari - Italy | 2 | ViolProvenceVP | | Catanese |  |
| CT-Clone B1 | Bari - Italy | 2 | B1 | | Catanese |  |
| CT-clone B5 | Bari - Italy | 2 | B2 | | Catanese |  |
| CT-Clone B7 | Bari - Italy | 2 | B7 | | Catanese |  |
| CT-Niscemese | Bari - Italy | 2 | NiscemeseCT | | Catanese |  |
| CT-Violet Provence ViolMarg | Bari - Italy | 2 | ViolProvenceVM | | Catanese |  |
| CT-Violet Provence VP41 | Bari - Italy | 2 | ViolProvence41I | | Catanese |  |
| CT-Violet Provence VP45 | Bari - Italy | 2 | ViolProvence45I | | Catanese |  |
| CT-Violet Provence VP73 | Bari - Italy | 2 | ViolProvence73 | | Catanese |  |
| CT-Violetto di Sicilia clone 13/2 | Bari - Italy | 2 | | ViolettoSicilia13 | Catanese | |
| CT-Violetto di Sicilia clone 14/9 | Bari - Italy | 2 | ViolettoSicilia14 | | Catanese |  |
| CT-Violetto di Sicilia clone 3/10 | Bari - Italy | 2 | ViolettoSicilia10 | | Catanese |  |
| CT-Violetto di Sicilia clone 3/6 | Bari - Italy | 2 | ViolettoSicilia3 | | Catanese |  |
| CT-Violetto di Sicilia clone 4/8 | Bari - Italy | 2 | ViolettoSicilia4 | | Catanese |  |
| CT-Violetto di Sicilia clone 6/1 | Bari - Italy | 2 | ViolettoSicilia61 | | Catanese |  |
| CT-Violetto di Sicilia clone 6/4 | Bari - Italy | 2 | ViolettoSicilia64 | | Catanese |  |
| CT-Violetto di Sicilia clone 9/8 | Bari - Italy | 2 | ViolettoSicilia98 | | Catanese |  |
| CT-Violetto di Sicilia clone B3 | Bari - Italy | 2 | ViolettoSiciliab3 | | Catanese |  |
| CT-Violetto di Sicilia clone I2 | Bari - Italy | 2 | ViolettoSiciliai2 | | Catanese |  |
| CT-Violetto di Sicilia clone I3 | Bari - Italy | 2 | ViolettoSiciliai3 | | Catanese |  |
| CT-ViolProvence Viol pop | Bari - Italy | 2 | ViolProvenceV | | Catanese |  |
| CT-ViolProvence VP Gela | Bari - Italy | 2 | ViolProvenceVPG | | Catanese |  |
| CT-ViolProvence VPRosolini | Bari - Italy | 2 | ViolProvenceVR | | Catanese |  |
| Escarot | France | 5 | Escarot | | Catanese |  |
| Gagliardo Sgrò | Bari - Italy | 2 | GagliardoSgrò | | Catanese |  |
| Mased’u | Spain | 2 | Masedu | | Catanese |  |
| Mola MO-3 | Bari - Italy | 13 | Mola | | Catanese |  |
| Niscemese | Bari - Italy | 2 | NiscemeseBA | | Catanese |  |
| Violet de Provence | France | 5 | VioletProvenceF | | Catanese |  |
| Violetto di Provenza BR-3 | Bari - Italy | 5 | VioletProvenceI | | Catanese |  |
| VP 45 | France | 5 | VioletProvence45 | | Catanese |  |
| VP-41 | Spain | 2 | VioletProvence41S | | Catanese |  |
| Aquara | Bari - Italy | 1 | Aquara | | OFF |  |
| Bianco di Ostuni LE-10 | Bari - Italy | 5 | BiancoOstuni | | OFF |  |
| Calice | France | 6 | Calice | | OFF |  |
| Carlit | Spain | 2 | Carlit | | OFF |  |
| CT-Clone MC14 | Bari - Italy | 2 | MC14 | | OFF |  |
| CT-Clone MC6 | Bari - Italy | 2 | MC6 | | OFF |  |
| CT-Clone MO10 | Bari - Italy | 2 | MO10 | | OFF |  |
| CT-Clone MO5 | Bari - Italy | 2 | MO5 | | OFF |  |
| CT-Clone MT1 | Bari - Italy | 2 | MT1 | | OFF |  |
| CT-CloneMC12 | Bari - Italy | 2 | MC12 | | OFF |  |
| CT-MOTTA non spinoso | Bari - Italy | 2 | Motta | | OFF |  |
| France | Spain | 2 | France | | OFF |  |
| Francesco | Spain | 2 | Francesco | | OFF |  |
| Gros vert de Laon | France | 5 | GrosVertdeLaon | | OFF |  |
| Italiana | Spain | 2 | Italiana | | OFF |  |
| Locale di Calimera LE-2 | Bari - Italy | 5 | Calimera | | OFF |  |
| Nero di Castrignano LE-1 | Bari - Italy | 5 | NeroCastrignano | | OFF |  |
| Nero di Ostuni LE-11 | Bari - Italy | 5 | NeroOstuni | | OFF |  |
| Vert de Provence | France | 5 | VertProvence | | OFF |  |
| Apolo | Spain | 2 | Apollo | | Romanesco |  |
| Ascolano | Viterbo - Italy | 8 | Ascolano | | Romanesco |  |
| B:H:-8 | Spain | 2 | CamusBretagneBH8 | | Romanesco |  |
| Bianco di Pertosa | Bari - Italy | 3 | BiancoPertosa | | Romanesco |  |
| C-3 | Spain | 1 | C3 | | Romanesco |  |
| Calico | France | 5 | Calico | | Romanesco |  |
| Camard | France | 5 | Camard | | Romanesco |  |
| Camerys Sp | Spain | 2 | CamerysS | | Romanesco |  |
| Campagnano | Spain | 2 | CampagnanoS | | Romanesco |  |
| Camus | France | 5 | Camus | | Romanesco |  |
| Camus Bretagne Sp | Spain | 2 | CamusBretagneS | | Romanesco |  |
| Capitan | France | 5 | Capitan | | Romanesco |  |
| Capuanella | Bari - Italy | 2 | Capuanella | | Romanesco |  |
| Carciofo di Pietrelcina | Bari - Italy | 2 | Pietrelcinab | | Romanesco |  |
| Caribou | France | 5 | Caribou | | Romanesco |  |
| Caribou Pt Sp | Spain | 2 | CaribouSp | | Romanesco |  |
| Castel | France | 5 | Castel | | Romanesco |  |
| CB | Spain | 2 | CalicoVerdeCB | | Romanesco |  |
| Compact | France | 5 | Compact | | Romanesco |  |
| CR | Spain | 2 | CalicoRojoCR | | Romanesco |  |
| Cric | France | 5 | Cric | | Romanesco |  |
| CT-Camard | Bari - Italy | 2 | CamardI | | Romanesco |  |
| CT-Camerys | Bari - Italy | 2 | CamerysI | | Romanesco |  |
| CT-Camus de Bretagne clone 6611 | Bari - Italy | 2 | CamusBretagneI | | Romanesco |  |
| CT-Romanesco clone1(rom7) | Bari - Italy | 4 | RomanescoCT | | Romanesco |  |
| IserniaCarciofo di Scapoli IS-1 | Bari - Italy | 4 | Isernia | | Romanesco |  |
| Jesino | Viterbo - Italy | 8 | Jesino | | Romanesco |  |
| Lira 3 | France | 5 | Lira | | Romanesco |  |
| Macau | Spain | 2 | Macau | | Romanesco |  |
| Montelupone A | Viterbo - Italy | 8 | MonteluponeA | | Romanesco |  |
| Montelupone B | Viterbo - Italy | 8 | MonteluponeB | | Romanesco |  |
| Moretto | Spain | 2 | Moretto | | Romanesco |  |
| Mut. Romanesco | Spain | 2 | MutRomanesco | | Romanesco |  |
| Ñato | Spain | 2 | Ñato | | Romanesco |  |
| Paestum | Viterbo - Italy | 8 | Paestum | | Romanesco |  |
| Pertosa | Viterbo - Italy | 8 | Pertosa | | Romanesco |  |
| Pètre | France | 5 | Pètre | | Romanesco |  |
| Pietralcina N1 | Bari - Italy | 2 | Pietralcina | | Romanesco |  |
| Pisa | Viterbo - Italy | 8 | Pisa | | Romanesco |  |
| Popvert | France | 5 | Popver | | Romanesco |  |
| Romain | France | 5 | Romain | | Romanesco |  |
| Romanesco | Bari - Italy | 2 | RomanescoBA | | Romanesco |  |
| Romanesco - Ancora Campagnano | Viterbo - Italy | 1 | Campagnano | | Romanesco |  |
| Romanesco - Ancora Grato I | Viterbo - Italy | 1 | GratoI | | Romanesco |  |
| Romanesco - Ancora S1 | Viterbo - Italy | 1 | S1 | | Romanesco |  |
| Romanesco - Ancora S10 | Viterbo - Italy | 1 | S10 | | Romanesco |  |
| Romanesco - Ancora S11 | Viterbo - Italy | 1 | S11 | | Romanesco |  |
| Romanesco - Ancora S13 | Viterbo - Italy | 1 | S13 | | Romanesco |  |
| Romanesco - Ancora S15 | Viterbo - Italy | 1 | S15 | | Romanesco |  |
| Romanesco - Ancora S16 | Viterbo - Italy | 1 | S16 | | Romanesco |  |
| Romanesco - Ancora S17 | Viterbo - Italy | 1 | S17 | | Romanesco |  |
| Romanesco - Ancora S18 | Viterbo - Italy | 1 | S18 | | Romanesco |  |
| Romanesco - Ancora S2 | Viterbo - Italy | 1 | S2 | | Romanesco |  |
| Romanesco - Ancora S20 | Viterbo - Italy | 1 | S20 | | Romanesco |  |
| Romanesco - Ancora S22 | Viterbo - Italy | 1 | S22 | | Romanesco |  |
| Romanesco - Ancora S23 | Viterbo - Italy | 1 | S23 | | Romanesco |  |
| Romanesco - Ancora S25 | Viterbo - Italy | 1 | S25 | | Romanesco |  |
| Romanesco - Ancora S26 | Viterbo - Italy | 1 | S26 | | Romanesco |  |
| Romanesco - Ancora S3 | Viterbo - Italy | 1 | S3 | | Romanesco |  |
| Romanesco - Ancora S30 | Viterbo - Italy | 1 | S30 | | Romanesco |  |
| Romanesco - Ancora S4 | Viterbo - Italy | 1 | S4 | | Romanesco |  |
| Romanesco - Ancora S5 | Viterbo - Italy | 1 | S5 | | Romanesco |  |
| Romanesco - Ancora S6 | Viterbo - Italy | 1 | S6 | | Romanesco |  |
| Romanesco - Troiani T31 | Viterbo - Italy | 1 | T31 | | Romanesco |  |
| Romanesco - Troiani T32 | Viterbo - Italy | 1 | T32 | | Romanesco |  |
| Romanesco - Troiani T33 | Viterbo - Italy | 1 | T33 | | Romanesco |  |
| Romanesco - Troiani T34 | Viterbo - Italy | 1 | T34 | | Romanesco |  |
| Romanesco - Troiani T35 | Viterbo - Italy | 1 | T35 | | Romanesco |  |
| Romanesco - Troiani T36 | Viterbo - Italy | 1 | T36 | | Romanesco |  |
| Romanesco - Troiani T37 | Viterbo - Italy | 1 | T37 | | Romanesco |  |
| Romanesco - Troiani T38 | Viterbo - Italy | 1 | T38 | | Romanesco |  |
| Romanesco - Troiani T39 | Viterbo - Italy | 1 | T39 | | Romanesco |  |
| Salambo | France | 5 | Salambo | | Romanesco |  |
| Salambo Sp | Spain | 2 | SalamboS | | Romanesco |  |
| Salanquet | France | 5 | Salanquet | | Romanesco |  |
| Salanquet Sp | Spain | 2 | SalanquetS | | Romanesco |  |
| Tondo di Paestum pt1 | Bari - Italy | 2 | TondoPaestum | | Romanesco |  |
| Tondo rosso di Paestum | Viterbo - Italy | 8 | TondoRossoPaestum | | Romanesco |  |
| Vertu | France | 5 | Vertu | | Romanesco |  |
| Criolla | Spain | 2 | Criolla | | Spiny |  |
| CT-Spinoso di Palermo clone1 | Bari - Italy | 3 | SpinosoPalermo | | Spiny |  |
| Hysponos | Spain | 2 | Hysponos | | Spiny |  |
| Spinoso Sardo | Spain | 2 | SpinosoSardo | | Spiny |  |
| Spinoso violetto di Luguria | Bari - Italy | 2 | SpinosoViolLiguria | | Spiny |  |
| Blanc hyérois | France | 5 | BlancHyérois | | Tudela |  |
| Blanca de Tudela clone 303 | Spain | 5 | Clon303 | | Tudela |  |
| Blanca de Tudela INIA B2 | Spain | 5 | INIA-B | | Tudela |  |
| Blanca de Tudela INIA D8 | Spain | 5 | INIA-D | | Tudela |  |
| Blanca de Tudela ITGA | Spain | 5 | ITGA | | Tudela |  |
| Blancal | France | 5 | Blancal | | Tudela |  |
| Cabeza de gato 1 BULL | Spain | 5 | Cabeza de gato | | Tudela |  |
| CT-Blancal Hyèrois | Bari - Italy | 2 | BlancHyèroisI | | Tudela |  |
| PAT-89 | Spain | 2 | PAT89 | | Tudela |  |
| CT-Violet Gapeau | Bari - Italy | 2 | VioletGapeauI | | Violet |  |
| Hydes Sp | Spain | 2 | Hydes | | Violet |  |
| S.Erasmo pt | Bari - Italy | 2 | SErasmo | | Violet |  |
| CT-Terom | Bari - Italy | 2 | TeromCT | | Violet |  |
| Terom LE-36 | Bari - Italy | 5 | TeromBA | | Violet |  |
| Velours | France | 5 | Velours | | Violet |  |
| Violet de Camargue | France | 5 | VioletCamargue | | Violet |  |
| Violet du Gapeau | France | 5 | VioletGapeau | | Violet |  |
| Violetto di Maremma | Bari - Italy | 4 | ViolettoMaremma | | Violet |  |
| Violetto di Toscana | Bari - Italy | 2 | ViolettoToscana | | Violet |  |
