# Supplementary material for: Genetic diversity and accession structure in European Cynara cardunculus collections
Source: PLoS One. 2017 Jun 1;12(6):e0178770. doi: 10.1371/journal.pone.0178770 (PMC5453587; doi:10.1371/journal.pone.0178770)
Supplement: S2 Table — (DOCX) [file pone.0178770.s004.docx]

S2 Table. ISSR primers and their annealing temperatures (T).

Primer Sequence 3’-5’ T (°C)

810 (GA)_8_T 43

818 (CA)_8_ G 54

827 (AC)_8_G 52

834 (AG)_8_YT 45

840 (GA)_8_YT 50

841 (GA) _8_YC 45

855 (CA) _8_RC 54

857 (AC) _8_YG 54

857C (AC) _8_YGC 59

857G (AC) _8_YGG 58

872 (GATA)_4_ 45

Y = Pyrimidine; R = Purine
