# Supplementary material for: Genetic diversity and accession structure in European Cynara cardunculus collections
Source: PLoS One. 2017 Jun 1;12(6):e0178770. doi: 10.1371/journal.pone.0178770 (PMC5453587; doi:10.1371/journal.pone.0178770)
Supplement: S3 Table — Number of bands or alleles in the case of SSR, frequency of the principal allele (MAF), Gene diversity and Expected Heterozygosity. (DOCX) [file pone.0178770.s005.docx]

S3 Table. Markers used to genotype 484 *Cynara cardunculus* var. *scolymus* genotypes with their genetic diversity parameters. Alleles in the case of SSR, frequency of the principal allele (MAF), Gene diversity and Expected Heterozygosity.

| **Marker** | **Alleles** | **MAF** | **He** | **PIC** | **Ho** | **Fis** | **Fit** | **Fst** | **F** |
| --- | --- | --- | --- | --- | --- | --- | --- | --- | --- |
| **AFLP** |  |  |  |  |  |  |  |  |  |
| EaccMcta |  | 0.816 | 0.235 | 0.186 |  |  |  |  |  |
| EacgMctt |  | 0.793 | 0.299 | 0.245 |  |  |  |  |  |
| EagcMctt |  | 0.853 | 0.198 | 0.159 |  |  |  |  |  |
| MacPca |  | 0.808 | 0.269 | 0.220 |  |  |  |  |  |
| MacPcg |  | 0.824 | 0.250 | 0.207 |  |  |  |  |  |
| MgcPca |  | 0.828 | 0.247 | 0.205 |  |  |  |  |  |
| MgcPcg |  | 0.834 | 0.241 | 0.201 |  |  |  |  |  |
| **Mean** |  | 0.822 | 0.248 | 0.203 |  |  |  |  |  |
| **ISSR** |  |  |  |  |  |  |  |  |  |
| 810 |  | 0.847 | 0.242 | 0.206 |  |  |  |  |  |
| 818 |  | 0.807 | 0.274 | 0.223 |  |  |  |  |  |
| 827 |  | 0.779 | 0.312 | 0.257 |  |  |  |  |  |
| 834 |  | 0.868 | 0.227 | 0.199 |  |  |  |  |  |
| 840 |  | 0.775 | 0.314 | 0.253 |  |  |  |  |  |
| 841 |  | 0.836 | 0.247 | 0.209 |  |  |  |  |  |
| 855 |  | 0.863 | 0.201 | 0.168 |  |  |  |  |  |
| 857 |  | 0.879 | 0.200 | 0.175 |  |  |  |  |  |
| 857c |  | 0.781 | 0.309 | 0.255 |  |  |  |  |  |
| 857g |  | 0.817 | 0.270 | 0.221 |  |  |  |  |  |
| 872 |  | 0.792 | 0.282 | 0.226 |  |  |  |  |  |
| **Mean** |  | 0.822 | 0.262 | 0.217 |  |  |  |  |  |
| **SSR** |  |  |  |  |  |  |  |  |  |
| CsCiCaCa05 | 8.0 | 0.862 | 0.253 | 0.247 | 0.228 | -0.333 | 0.140 | 0.355 | -0.293 |
| CDAT-01 | 6.0 | 0.357 | 0.743 | 0.701 | 0.857 | -0.993 | -0.112 | 0.442 | -0.994 |
| CLIB-02I | 7.0 | 0.266 | 0.812 | 0.785 | 0.411 | -0.696 | 0.413 | 0.654 | -0.689 |
| CLIB-02II | 4.0 | 0.962 | 0.074 | 0.072 | 0.037 | -1.000 | 0.728 | 0.864 | -1.000 |
| CLIB-12 | 3.0 | 0.397 | 0.660 | 0.586 | 0.890 | -0.995 | -0.325 | 0.336 | -0.996 |
| CMAFLP-01 | 3.0 | 0.794 | 0.343 | 0.310 | 0.390 | -0.986 | -0.001 | 0.496 | -0.982 |
| CMAFLP-04 | 8.0 | 0.671 | 0.503 | 0.459 | 0.117 | -0.738 | 0.802 | 0.886 | -0.671 |
| CMAFLP-05 | 1.0 | 1.000 | 0.000 | 0.000 | 0.000 | 0.000 | 1.000 | 1.000 | 0.000 |
| CMAFLP-18 | 3.0 | 0.569 | 0.500 | 0.385 | 0.783 | -0.994 | -0.516 | 0.240 | -0.988 |
| CMAL06 | 7.0 | 0.533 | 0.540 | 0.440 | 0.541 | -0.833 | -0.080 | 0.410 | -0.803 |
| CMAL-108 | 3.0 | 0.515 | 0.607 | 0.533 | 0.693 | -0.985 | -0.123 | 0.434 | -0.970 |
| CMAL11 | 3.0 | 0.668 | 0.444 | 0.346 | 0.472 | -0.612 | -0.067 | 0.338 | -0.561 |
| CMAL117 | 14.0 | 0.737 | 0.415 | 0.370 | 0.244 | -0.474 | 0.295 | 0.521 | -0.406 |
| CMAL21 | 8.0 | 0.654 | 0.513 | 0.462 | 0.512 | -0.813 | -0.069 | 0.411 | -0.770 |
| CMAL24 | 6.0 | 0.419 | 0.657 | 0.587 | 0.188 | -0.606 | 0.608 | 0.756 | -0.523 |
| CMAL-25 | 2.0 | 0.500 | 0.500 | 0.375 | 1.000 | -1.000 | -0.927 | 0.036 | -1.000 |
| CsPal02 | 10.0 | 0.684 | 0.477 | 0.426 | 0.343 | -0.672 | 0.235 | 0.543 | -0.608 |
| CsPal03 | 5.0 | 0.504 | 0.648 | 0.593 | 0.791 | -0.907 | -0.251 | 0.344 | -0.905 |
| CsEST03 | 4.0 | 0.684 | 0.463 | 0.398 | 0.408 | -0.356 | 0.081 | 0.322 | -0.329 |
| FA2-GAT | 4.0 | 0.891 | 0.195 | 0.177 | 0.214 | -0.806 | -0.081 | 0.401 | -0.747 |
| **Mean** | 5.5 | 0.633 | 0.467 | 0.413 | 0.456 | -0.740 | 0.087 | 0.489 | -0.712 |
| Overall mean |  | 0.723 | 0.367 | 0.318 |  |  |  |  |  |
