# Supplementary material for: Genetic diversity and accession structure in European Cynara cardunculus collections
Source: PLoS One. 2017 Jun 1;12(6):e0178770. doi: 10.1371/journal.pone.0178770 (PMC5453587; doi:10.1371/journal.pone.0178770)
Supplement: S4 Table — Number of bands or alleles in the case of SSR, frequency of the principal allele (MAF), Gene diversity and Expected Heterozygosity. (DOCX) [file pone.0178770.s006.docx]

S4 Table. Markers used to genotype 72 *Cynara cardunculus* var. *altilis* genotypes with their genetic diversity parameters. Alleles in the case of SSR, frequency of the principal allele (MAF), Gene diversity and Expected Heterozygosity.

| **Marker** | **Alleles** | **MAF** | **He** | **PIC** | **Ho** | **Fis** | **Fit** | **Fst** | **F** |
| --- | --- | --- | --- | --- | --- | --- | --- | --- | --- |
| **AFLP** |  |  |  |  |  |  |  |  |  |
| EaccMcta |  | 0.861 | 0.189 | 0.154 |  |  |  |  |  |
| EacgMctt |  | 0.979 | 0.036 | 0.031 |  |  |  |  |  |
| EagcMctt |  | 0.904 | 0.137 | 0.115 |  |  |  |  |  |
| MacPca |  | 0.827 | 0.243 | 0.199 |  |  |  |  |  |
| MacPcg |  | 0.848 | 0.219 | 0.182 |  |  |  |  |  |
| MgcPca |  | 0.875 | 0.177 | 0.146 |  |  |  |  |  |
| MgcPcg |  | 0.862 | 0.197 | 0.163 |  |  |  |  |  |
| **Mean** |  | 0.880 | 0.171 | 0.141 |  |  |  |  |  |
| **ISSR** |  |  |  |  |  |  |  |  |  |
| 810 |  | 0.853 | 0.191 | 0.154 |  |  |  |  |  |
| 818 |  | 0.897 | 0.132 | 0.108 |  |  |  |  |  |
| 827 |  | 0.887 | 0.176 | 0.148 |  |  |  |  |  |
| 834 |  | 1.000 | 0.000 | 0.000 |  |  |  |  |  |
| 840 |  | 0.885 | 0.169 | 0.139 |  |  |  |  |  |
| 841 |  | 0.972 | 0.045 | 0.038 |  |  |  |  |  |
| 855 |  | 0.770 | 0.328 | 0.265 |  |  |  |  |  |
| 857 |  | 0.878 | 0.191 | 0.163 |  |  |  |  |  |
| 857c |  | 0.766 | 0.322 | 0.256 |  |  |  |  |  |
| 857g |  | 0.842 | 0.239 | 0.196 |  |  |  |  |  |
| 872 |  | 0.776 | 0.303 | 0.241 |  |  |  |  |  |
| **Mean** |  | 0.866 | 0.190 | 0.155 |  |  |  |  |  |
| **SSR** |  |  |  |  |  |  |  |  |  |
| CsCiCaCa05 | 6 | 0.592 | 0.546 | 0.473 | 0.354 | -0.099 | 0.360 | 0.417 | -0.092 |
| CDAT-01 | 5 | 0.543 | 0.527 | 0.422 | 0.471 | -0.183 | 0.091 | 0.231 | -0.138 |
| CLIB-02I | 3 | 0.799 | 0.339 | 0.308 | 0.254 | -0.056 | 0.274 | 0.312 | -0.023 |
| CLIB-02II | 3 | 0.507 | 0.621 | 0.551 | 0.130 | 0.631 | 0.791 | 0.433 | 0.614 |
| CLIB-12 | 3 | 0.934 | 0.126 | 0.122 | 0.132 | -0.174 | -0.053 | 0.103 | -0.145 |
| CMAFLP-01 | 5 | 0.414 | 0.667 | 0.600 | 0.259 | 0.116 | 0.629 | 0.581 | 0.103 |
| CMAFLP-04 | 10 | 0.352 | 0.800 | 0.777 | 0.222 | 0.249 | 0.839 | 0.786 | 0.280 |
| CMAFLP-05 | 1 | 1.000 | 0.000 | 0.000 | 0.000 | 0.000 | 0.000 | 0.000 | 0 |
| CMAFLP-18 | 10 | 0.294 | 0.833 | 0.814 | 0.508 | -0.012 | 0.375 | 0.382 | -0.041 |
| CMAL06 | 9 | 0.431 | 0.719 | 0.680 | 0.462 | -0.068 | 0.344 | 0.386 | -0.066 |
| CMAL-108 | 4 | 0.881 | 0.218 | 0.209 | 0.179 | -0.243 | 0.190 | 0.348 | -0.223 |
| CMAL11 | 2 | 0.738 | 0.386 | 0.312 | 0.523 | -0.639 | -0.353 | 0.175 | -0.522 |
| CMAL117 | 7 | 0.738 | 0.416 | 0.374 | 0.215 | 0.191 | 0.450 | 0.320 | 0.128 |
| CMAL21 | 8 | 0.469 | 0.727 | 0.701 | 0.631 | -0.212 | 0.137 | 0.288 | -0.172 |
| CMAL24 | 2 | 0.887 | 0.200 | 0.180 | 0.000 | 1.000 | 1.000 | 0.349 | 1.000 |
| CMAL-25 | 2 | 0.529 | 0.498 | 0.374 | 0.941 | -0.943 | -0.909 | 0.018 | -0.932 |
| CsPal02 | 8 | 0.546 | 0.641 | 0.603 | 0.123 | 0.689 | 0.819 | 0.419 | 0.656 |
| CsPal03 | 3 | 0.569 | 0.515 | 0.410 | 0.215 | 0.153 | 0.560 | 0.481 | 0.201 |
| CsEST03 | 7 | 0.531 | 0.609 | 0.544 | 0.369 | 0.068 | 0.427 | 0.385 | 0.085 |
| FA2-GAT | 2 | 0.662 | 0.448 | 0.348 | 0.462 | -0.603 | -0.046 | 0.347 | -0.520 |
| **Mean** | 5.0 | 0.621 | 0.492 | 0.440 | 0.323 | -0.007 | 0.296 | 0.338 | 0.010 |
| Overall mean |  | 0.739 | 0.345 | 0.303 |  |  |  |  |  |
