# Supplementary material for: Genetic diversity and accession structure in European Cynara cardunculus collections
Source: PLoS One. 2017 Jun 1;12(6):e0178770. doi: 10.1371/journal.pone.0178770 (PMC5453587; doi:10.1371/journal.pone.0178770)
Supplement: S5 Table — (DOCX) [file pone.0178770.s007.docx]

S5 Table. Significant linkage disequilibrium (significance level=0.0500) between loci indicated by +.

| Locus # | CMAL11 | CMAL117 | CMAL24 | CsCaCa05 | CsEST03 | FA2-GAT3 | CMAL21 | CMAL06 | CsPal03 | CsPal02 | CDAT-01 | CMAL-108 | CLIB-02I | CLIB-02II | CLIB-12 | CMAL-25 | CMAFLP-05 | CMAFLP-01 | CMAFLP-04 | CMAFLP-18 |
| --- | --- | --- | --- | --- | --- | --- | --- | --- | --- | --- | --- | --- | --- | --- | --- | --- | --- | --- | --- | --- |
| CMAL11 | ***** | **+** | **+** | **+** | **+** | **+** | **+** | **+** | **+** | **+** | **+** | **+** | **+** | **+** | **+** | **-** | **-** | **+** | **+** | **+** |
| CMAL117 | **+** | ***** | **+** | **+** | **+** | **+** | **+** | **+** | **+** | **+** | **+** | **+** | **+** | **+** | **+** | **-** | **-** | **+** | **+** | **+** |
| CMAL24 | **+** | **+** | ***** | **+** | **+** | **+** | **+** | **+** | **+** | **+** | **+** | **+** | **+** | **+** | **+** | **-** | **-** | **+** | **+** | **+** |
| CsCaCa05 | **+** | **+** | **+** | ***** | **+** | **+** | **+** | **+** | **+** | **+** | **+** | **+** | **+** | **+** | **+** | **-** | **-** | **+** | **+** | **+** |
| CsEST03 | **+** | **+** | **+** | **+** | ***** | **+** | **+** | **+** | **+** | **+** | **+** | **+** | **+** | **+** | **+** | **-** | **-** | **+** | **+** | **+** |
| FA2-GAT3 | **+** | **+** | **+** | **+** | **+** | ***** | **+** | **+** | **+** | **+** | **+** | **+** | **+** | **+** | **+** | **+** | **-** | **+** | **+** | **+** |
| CMAL21 | **+** | **+** | **+** | **+** | **+** | **+** | ***** | **+** | **+** | **+** | **+** | **+** | **+** | **+** | **+** | **+** | **-** | **+** | **+** | **+** |
| CMAL06 | **+** | **+** | **+** | **+** | **+** | **+** | **+** | ***** | **+** | **+** | **+** | **+** | **+** | **+** | **+** | **+** | **-** | **+** | **+** | **+** |
| CsPal03 | **+** | **+** | **+** | **+** | **+** | **+** | **+** | **+** | ***** | **+** | **+** | **+** | **+** | **+** | **+** | **+** | **-** | **+** | **+** | **+** |
| CsPal02 | **+** | **+** | **+** | **+** | **+** | **+** | **+** | **+** | **+** | ***** | **+** | **+** | **+** | **+** | **+** | **-** | **-** | **+** | **+** | **+** |
| CDAT-01 | **+** | **+** | **+** | **+** | **+** | **+** | **+** | **+** | **+** | **+** | ***** | **+** | **+** | **+** | **+** | **+** | **-** | **+** | **+** | **+** |
| CMAL-108 | **+** | **+** | **+** | **+** | **+** | **+** | **+** | **+** | **+** | **+** | **+** | ***** | **+** | **+** | **+** | **+** | **-** | **+** | **+** | **+** |
| CLIB-02I | **+** | **+** | **+** | **+** | **+** | **+** | **+** | **+** | **+** | **+** | **+** | **+** | ***** | **+** | **+** | **+** | **-** | **+** | **+** | **+** |
| CLIB-02II | **+** | **+** | **+** | **+** | **+** | **+** | **+** | **+** | **+** | **+** | **+** | **+** | **+** | ***** | **+** | **+** | **-** | **+** | **+** | **+** |
| CLIB-12 | **+** | **+** | **+** | **+** | **+** | **+** | **+** | **+** | **+** | **+** | **+** | **+** | **+** | **+** | ***** | **+** | **-** | **+** | **+** | **+** |
| CMAL-25 | **-** | **-** | **-** | **-** | **-** | **+** | **+** | **+** | **+** | **-** | **+** | **+** | **+** | **+** | **+** | ***** | **-** | **+** | **+** | **+** |
| CMAFLP-05 | **-** | **-** | **-** | **-** | **-** | **-** | **-** | **-** | **-** | **-** | **-** | **-** | **-** | **-** | **-** | **-** | ***** | **-** | **-** | **-** |
| CMAFLP-01 | **+** | **+** | **+** | **+** | **+** | **+** | **+** | **+** | **+** | **+** | **+** | **+** | **+** | **+** | **+** | **+** | **-** | ***** | **+** | **+** |
| CMAFLP-04 | **+** | **+** | **+** | **+** | **+** | **+** | **+** | **+** | **+** | **+** | **+** | **+** | **+** | **+** | **+** | **+** | **-** | **+** | ***** | **+** |
| CMAFLP-18 | **+** | **+** | **+** | **+** | **+** | **+** | **+** | **+** | **+** | **+** | **+** | **+** | **+** | **+** | **+** | **+** | **-** | **+** | **+** | ***** |
