# Supplementary material for: Genetic diversity and accession structure in European Cynara cardunculus collections
Source: PLoS One. 2017 Jun 1;12(6):e0178770. doi: 10.1371/journal.pone.0178770 (PMC5453587; doi:10.1371/journal.pone.0178770)
Supplement: S6 Table — Polymorphism (P), expected heterozygosity (He), observed heterozygosity (Ho), Fixation Index (F), Number of alleles with frequency higher than 5% (Na), Shannon's Information Index (I), and number of less common Alleles (< = 25%) (No). (DOCX) [file pone.0178770.s008.docx]

S6 Table. Accession diversity measured with dominant (D) or codominant (CD) markers. Polymorphism (P), expected heterozygosity (He), observed heterozygosity (Ho), Fixation Index (F), Number of alleles with frequency higher than 5% (Na), Shannon's Information Index (I), and number of less common Alleles (<=25%) (No).

| **Abbreviation** | **P** | | **He** | | **Ho** | **F** | **Na** | **I** | | **No.** |
| --- | --- | --- | --- | --- | --- | --- | --- | --- | --- | --- |
|  | D | CD | D | CD | CD | CD | CD | D | CD | CD |
| Apollo | 0.204 | 0.400 | 0.085 | 0.200 | 0.400 | -1.000 | 1.100 | 0.124 | 0.277 | 0.050 |
| Aquara | 0.000 | 0.350 | 0.000 | 0.175 | 0.350 | -1.000 | 1.350 | 0.000 | 0.243 | 0.050 |
| Ascolano | 0.424 | 0.700 | 0.122 | 0.339 | 0.605 | -0.715 | 1.800 | 0.190 | 0.489 | 0.050 |
| B1 | 0.092 | 0.550 | 0.038 | 0.281 | 0.500 | -0.782 | 1.600 | 0.056 | 0.399 | 0.100 |
| B2 | 0.174 | 0.550 | 0.072 | 0.275 | 0.550 | -1.000 | 1.550 | 0.105 | 0.381 | 0.100 |
| B7 | 0.080 | 0.550 | 0.033 | 0.269 | 0.525 | -0.939 | 1.550 | 0.048 | 0.375 | 0.000 |
| BiancoOstuni | 0.336 | 0.750 | 0.124 | 0.393 | 0.670 | -0.727 | 2.150 | 0.184 | 0.607 | 0.900 |
| BiancoPertosa | 0.215 | 0.400 | 0.080 | 0.200 | 0.400 | -1.000 | 1.400 | 0.120 | 0.277 | 0.350 |
| Blancal | 0.491 | 0.700 | 0.148 | 0.346 | 0.543 | -0.584 | 1.850 | 0.231 | 0.506 | 0.250 |
| BlancHyérois | 0.263 | 0.550 | 0.085 | 0.254 | 0.323 | -0.286 | 1.650 | 0.130 | 0.377 | 0.200 |
| BlancHyèroisI | 0.142 | 0.450 | 0.059 | 0.219 | 0.425 | -0.926 | 1.450 | 0.086 | 0.305 | 0.200 |
| BlancodeHuerva | 0.315 | 0.700 | 0.109 | 0.338 | 0.400 | -0.146 | 2.000 | 0.164 | 0.530 | 0.900 |
| BlancoPeralta | 0.306 | 0.850 | 0.106 | 0.369 | 0.408 | -0.097 | 2.200 | 0.159 | 0.588 | 0.800 |
| BlancoValencia | 0.253 | 0.600 | 0.087 | 0.273 | 0.318 | -0.136 | 1.800 | 0.131 | 0.426 | 0.700 |
| Brindisi | 0.261 | 0.600 | 0.108 | 0.294 | 0.525 | -0.778 | 1.600 | 0.158 | 0.409 | 0.000 |
| Brindisino | 0.502 | 0.650 | 0.145 | 0.304 | 0.471 | -0.508 | 1.900 | 0.223 | 0.464 | 0.450 |
| C3 | 0.000 | 0.100 | 0.000 | 0.050 | 0.100 | -1.000 | 0.650 | 0.000 | 0.069 | 0.000 |
| Cabeza de gato | 0.169 | 0.600 | 0.061 | 0.305 | 0.530 | -0.747 | 1.650 | 0.091 | 0.432 | 0.250 |
| Cacique | 0.402 | 0.750 | 0.130 | 0.361 | 0.542 | -0.508 | 1.850 | 0.200 | 0.526 | 0.150 |
| Calice | 0.554 | 0.850 | 0.165 | 0.346 | 0.500 | -0.313 | 2.050 | 0.256 | 0.529 | 0.150 |
| Calico | 0.475 | 0.450 | 0.143 | 0.182 | 0.240 | -0.205 | 1.500 | 0.224 | 0.274 | 0.150 |
| CalicoRojoCR | 0.149 | 0.350 | 0.062 | 0.169 | 0.325 | -0.905 | 1.350 | 0.090 | 0.236 | 0.100 |
| CalicoVerdeCB | 0.159 | 0.450 | 0.066 | 0.200 | 0.350 | -0.704 | 1.400 | 0.096 | 0.286 | 0.000 |
| Calimera | 0.284 | 0.450 | 0.106 | 0.228 | 0.420 | -0.845 | 1.600 | 0.157 | 0.341 | 0.300 |
| Camard | 0.489 | 0.700 | 0.153 | 0.329 | 0.610 | -0.808 | 1.750 | 0.236 | 0.472 | 0.300 |
| CamardI | 0.079 | 0.500 | 0.033 | 0.244 | 0.475 | -0.933 | 1.450 | 0.048 | 0.340 | 0.250 |
| CamerysI | 0.134 | 0.500 | 0.056 | 0.244 | 0.425 | -0.747 | 1.550 | 0.081 | 0.351 | 0.300 |
| CamerysS | 0.095 | 0.400 | 0.040 | 0.194 | 0.375 | -0.917 | 1.400 | 0.058 | 0.271 | 0.200 |
| Campagnano | 0.000 | 0.450 | 0.000 | 0.225 | 0.450 | -1.000 | 1.450 | 0.000 | 0.312 | 0.000 |
| CampagnanoS | 0.097 | 0.600 | 0.040 | 0.288 | 0.550 | -0.889 | 1.600 | 0.058 | 0.403 | 0.000 |
| Camus | 0.501 | 0.450 | 0.162 | 0.230 | 0.420 | -0.848 | 1.500 | 0.248 | 0.328 | 0.200 |
| CamusBretagneBH8 | 0.131 | 0.350 | 0.054 | 0.169 | 0.275 | -0.619 | 1.350 | 0.079 | 0.236 | 0.100 |
| CamusBretagneI | 0.101 | 0.550 | 0.042 | 0.263 | 0.500 | -0.879 | 1.550 | 0.061 | 0.368 | 0.200 |
| CamusBretagneS | 0.120 | 0.350 | 0.050 | 0.163 | 0.300 | -0.810 | 1.150 | 0.073 | 0.230 | 0.100 |
| Capitan | 0.480 | 0.600 | 0.149 | 0.241 | 0.410 | -0.587 | 1.700 | 0.230 | 0.365 | 0.250 |
| Capuanella | 0.171 | 0.500 | 0.071 | 0.250 | 0.500 | -1.000 | 1.500 | 0.104 | 0.347 | 0.000 |
| Caribou | 0.294 | 0.400 | 0.100 | 0.166 | 0.303 | -0.710 | 1.400 | 0.151 | 0.239 | 0.200 |
| CaribouSp | 0.094 | 0.400 | 0.039 | 0.188 | 0.325 | -0.700 | 1.450 | 0.057 | 0.275 | 0.250 |
| Carlit | 0.237 | 0.400 | 0.098 | 0.194 | 0.375 | -0.917 | 1.350 | 0.143 | 0.271 | 0.000 |
| Castel | 0.504 | 0.400 | 0.158 | 0.180 | 0.340 | -0.817 | 1.400 | 0.244 | 0.255 | 0.250 |
| Catanese | 0.090 | 0.600 | 0.037 | 0.288 | 0.550 | -0.889 | 1.600 | 0.055 | 0.403 | 0.100 |
| Chrysanthème | 0.494 | 0.600 | 0.154 | 0.263 | 0.458 | -0.660 | 1.700 | 0.238 | 0.391 | 0.150 |
| ChrysanthèmeS | 0.105 | 0.450 | 0.043 | 0.231 | 0.450 | -0.956 | 1.500 | 0.063 | 0.329 | 0.150 |
| Clon303 | 0.198 | 0.700 | 0.071 | 0.322 | 0.540 | -0.633 | 1.750 | 0.107 | 0.463 | 0.300 |
| Compact | 0.467 | 0.400 | 0.147 | 0.163 | 0.270 | -0.561 | 1.400 | 0.226 | 0.235 | 0.100 |
| Cric | 0.474 | 0.450 | 0.158 | 0.213 | 0.330 | -0.496 | 1.500 | 0.240 | 0.307 | 0.200 |
| Criolla | 0.146 | 0.400 | 0.060 | 0.188 | 0.350 | -0.833 | 1.400 | 0.088 | 0.264 | 0.100 |
| DelCortijo | 0.362 | 0.500 | 0.137 | 0.185 | 0.183 | 0.024 | 1.650 | 0.202 | 0.295 | 0.650 |
| Escarot | 0.489 | 0.800 | 0.151 | 0.335 | 0.380 | -0.128 | 1.900 | 0.234 | 0.503 | 0.150 |
| France | 0.089 | 0.500 | 0.037 | 0.244 | 0.425 | -0.733 | 1.500 | 0.054 | 0.340 | 0.300 |
| Francesco | 0.138 | 0.500 | 0.057 | 0.250 | 0.500 | -1.000 | 1.500 | 0.083 | 0.347 | 0.050 |
| GagliardoSgrò | 0.168 | 0.550 | 0.070 | 0.269 | 0.525 | -0.939 | 1.550 | 0.102 | 0.375 | 0.050 |
| GratoI | 0.000 | 0.500 | 0.000 | 0.250 | 0.500 | -1.000 | 1.450 | 0.000 | 0.347 | 0.100 |
| GrosVertdeLaon | 0.379 | 0.700 | 0.134 | 0.293 | 0.450 | -0.418 | 1.750 | 0.201 | 0.431 | 0.250 |
| Hydes | 0.176 | 0.200 | 0.073 | 0.094 | 0.150 | -0.567 | 0.900 | 0.106 | 0.143 | 0.100 |
| Hysponos | 0.215 | 0.350 | 0.089 | 0.169 | 0.325 | -0.905 | 1.350 | 0.130 | 0.236 | 0.150 |
| INIA-B | 0.239 | 0.650 | 0.087 | 0.298 | 0.520 | -0.694 | 1.650 | 0.129 | 0.420 | 0.250 |
| INIA-D | 0.184 | 0.850 | 0.062 | 0.420 | 0.620 | -0.434 | 2.100 | 0.095 | 0.627 | 0.450 |
| Isernia | 0.259 | 0.500 | 0.092 | 0.233 | 0.400 | -0.643 | 1.550 | 0.138 | 0.337 | 0.400 |
| Italiana | 0.106 | 0.400 | 0.044 | 0.188 | 0.300 | -0.583 | 1.400 | 0.064 | 0.264 | 0.200 |
| ITGA | 0.085 | 0.800 | 0.035 | 0.373 | 0.570 | -0.505 | 1.900 | 0.052 | 0.539 | 0.400 |
| Jesino | 0.366 | 0.600 | 0.106 | 0.263 | 0.381 | -0.355 | 1.700 | 0.165 | 0.388 | 0.350 |
| Lira | 0.497 | 0.600 | 0.156 | 0.259 | 0.420 | -0.525 | 1.650 | 0.241 | 0.378 | 0.150 |
| LlenoEspaña | 0.283 | 0.750 | 0.097 | 0.319 | 0.353 | -0.088 | 2.050 | 0.146 | 0.507 | 1.000 |
| Lumbier | 0.296 | 0.600 | 0.104 | 0.233 | 0.220 | 0.026 | 1.700 | 0.156 | 0.365 | 0.650 |
| Macau | 0.108 | 0.350 | 0.045 | 0.163 | 0.300 | -0.810 | 1.350 | 0.065 | 0.230 | 0.100 |
| Masedu | 0.110 | 0.550 | 0.046 | 0.275 | 0.550 | -1.000 | 1.550 | 0.066 | 0.381 | 0.100 |
| MC12 | 0.085 | 0.600 | 0.035 | 0.294 | 0.575 | -0.944 | 1.600 | 0.052 | 0.409 | 0.200 |
| MC14 | 0.100 | 0.650 | 0.041 | 0.319 | 0.575 | -0.795 | 1.650 | 0.060 | 0.444 | 0.200 |
| MC6 | 0.187 | 0.550 | 0.077 | 0.269 | 0.525 | -0.939 | 1.550 | 0.113 | 0.375 | 0.100 |
| MO10 | 0.124 | 0.600 | 0.051 | 0.300 | 0.550 | -0.833 | 1.600 | 0.075 | 0.416 | 0.150 |
| MO5 | 0.185 | 0.650 | 0.077 | 0.313 | 0.600 | -0.897 | 1.650 | 0.112 | 0.437 | 0.150 |
| Mola | 0.449 | 0.650 | 0.143 | 0.292 | 0.481 | -0.584 | 1.750 | 0.217 | 0.434 | 0.250 |
| MonteluponeA | 0.435 | 0.550 | 0.140 | 0.235 | 0.377 | -0.552 | 1.600 | 0.213 | 0.345 | 0.200 |
| MonteluponeB | 0.401 | 0.500 | 0.118 | 0.236 | 0.356 | -0.505 | 1.650 | 0.183 | 0.357 | 0.250 |
| Moretto | 0.130 | 0.350 | 0.054 | 0.175 | 0.350 | -1.000 | 1.350 | 0.079 | 0.243 | 0.100 |
| Motta | 0.154 | 0.400 | 0.064 | 0.194 | 0.275 | -0.417 | 1.400 | 0.093 | 0.271 | 0.100 |
| MT1 | 0.198 | 0.650 | 0.082 | 0.300 | 0.525 | -0.713 | 1.700 | 0.120 | 0.435 | 0.100 |
| MutRomanesco | 0.119 | 0.350 | 0.049 | 0.169 | 0.275 | -0.619 | 0.900 | 0.072 | 0.236 | 0.050 |
| NeroCastrignano | 0.266 | 0.500 | 0.100 | 0.216 | 0.390 | -0.703 | 1.600 | 0.148 | 0.324 | 0.250 |
| NeroOstuni | 0.276 | 0.500 | 0.102 | 0.235 | 0.360 | -0.490 | 1.650 | 0.151 | 0.355 | 0.450 |
| NiscemeseBA | 0.071 | 0.500 | 0.029 | 0.250 | 0.450 | -0.813 | 1.550 | 0.043 | 0.357 | 0.100 |
| NiscemeseCT | 0.120 | 0.500 | 0.050 | 0.244 | 0.475 | -0.933 | 1.500 | 0.073 | 0.340 | 0.000 |
| Ñato | 0.114 | 0.450 | 0.047 | 0.219 | 0.425 | -0.926 | 1.400 | 0.069 | 0.305 | 0.250 |
| Paestum | 0.389 | 0.700 | 0.115 | 0.315 | 0.513 | -0.577 | 1.800 | 0.178 | 0.458 | 0.150 |
| PAT89 | 0.120 | 0.550 | 0.050 | 0.269 | 0.525 | -0.939 | 1.550 | 0.073 | 0.375 | 0.100 |
| Pertosa | 0.426 | 0.500 | 0.124 | 0.220 | 0.369 | -0.537 | 1.550 | 0.193 | 0.320 | 0.300 |
| Pètre | 0.240 | 0.600 | 0.078 | 0.238 | 0.300 | -0.131 | 1.650 | 0.119 | 0.355 | 0.150 |
| Pietralcina | 0.140 | 0.700 | 0.058 | 0.325 | 0.500 | -0.524 | 1.700 | 0.084 | 0.459 | 0.150 |
| Pietrelcinab | 0.138 | 0.500 | 0.057 | 0.250 | 0.500 | -1.000 | 1.500 | 0.083 | 0.347 | 0.100 |
| Pisa | 0.417 | 0.500 | 0.119 | 0.212 | 0.369 | -0.585 | 1.500 | 0.186 | 0.303 | 0.100 |
| PleinBlancInerme | 0.295 | 0.550 | 0.108 | 0.191 | 0.138 | 0.237 | 1.700 | 0.161 | 0.311 | 0.650 |
| Popver | 0.493 | 0.350 | 0.161 | 0.153 | 0.270 | -0.696 | 1.350 | 0.245 | 0.218 | 0.000 |
| PuvisAmélioré | 0.302 | 0.800 | 0.103 | 0.339 | 0.374 | -0.094 | 2.050 | 0.155 | 0.533 | 0.900 |
| RojoAgreda | 0.372 | 0.600 | 0.131 | 0.261 | 0.230 | 0.142 | 1.800 | 0.197 | 0.417 | 0.850 |
| Romain | 0.440 | 0.550 | 0.147 | 0.274 | 0.500 | -0.806 | 1.600 | 0.223 | 0.389 | 0.100 |
| RomanescoBA | 0.187 | 0.700 | 0.067 | 0.328 | 0.625 | -0.863 | 1.700 | 0.101 | 0.461 | 0.050 |
| RomanescoCT | 0.185 | 0.650 | 0.077 | 0.325 | 0.650 | -1.000 | 1.650 | 0.112 | 0.451 | 0.100 |
| RougeAlger | 0.373 | 0.850 | 0.131 | 0.421 | 0.423 | -0.022 | 2.250 | 0.196 | 0.657 | 0.850 |
| S1 | 0.000 | 0.450 | 0.000 | 0.225 | 0.450 | -1.000 | 1.350 | 0.000 | 0.312 | 0.000 |
| S10 | 0.000 | 0.500 | 0.000 | 0.250 | 0.500 | -1.000 | 1.500 | 0.000 | 0.347 | 0.050 |
| S11 | 0.000 | 0.500 | 0.000 | 0.250 | 0.500 | -1.000 | 1.500 | 0.000 | 0.347 | 0.000 |
| S13 | 0.000 | 0.500 | 0.000 | 0.250 | 0.500 | -1.000 | 1.500 | 0.000 | 0.347 | 0.000 |
| S15 | 0.000 | 0.550 | 0.000 | 0.275 | 0.550 | -1.000 | 1.550 | 0.000 | 0.381 | 0.000 |
| S16 | 0.000 | 0.450 | 0.000 | 0.225 | 0.450 | -1.000 | 1.350 | 0.000 | 0.312 | 0.000 |
| S17 | 0.000 | 0.550 | 0.000 | 0.275 | 0.550 | -1.000 | 1.550 | 0.000 | 0.381 | 0.000 |
| S18 | 0.000 | 0.500 | 0.000 | 0.250 | 0.500 | -1.000 | 1.500 | 0.000 | 0.347 | 0.000 |
| S2 | 0.000 | 0.500 | 0.000 | 0.250 | 0.500 | -1.000 | 1.450 | 0.000 | 0.347 | 0.000 |
| S20 | 0.000 | 0.550 | 0.000 | 0.275 | 0.550 | -1.000 | 1.550 | 0.000 | 0.381 | 0.000 |
| S22 | 0.000 | 0.500 | 0.000 | 0.250 | 0.500 | -1.000 | 1.500 | 0.000 | 0.347 | 0.050 |
| S23 | 0.000 | 0.500 | 0.000 | 0.250 | 0.500 | -1.000 | 1.500 | 0.000 | 0.347 | 0.050 |
| S25 | 0.000 | 0.450 | 0.000 | 0.225 | 0.450 | -1.000 | 1.450 | 0.000 | 0.312 | 0.050 |
| S26 | 0.000 | 0.400 | 0.000 | 0.200 | 0.400 | -1.000 | 1.400 | 0.000 | 0.277 | 0.050 |
| S3 | 0.000 | 0.500 | 0.000 | 0.250 | 0.500 | -1.000 | 1.450 | 0.000 | 0.347 | 0.000 |
| S30 | 0.000 | 0.600 | 0.000 | 0.300 | 0.600 | -1.000 | 1.600 | 0.000 | 0.416 | 0.050 |
| S4 | 0.000 | 0.400 | 0.000 | 0.200 | 0.400 | -1.000 | 1.400 | 0.000 | 0.277 | 0.000 |
| S5 | 0.000 | 0.600 | 0.000 | 0.300 | 0.600 | -1.000 | 1.600 | 0.000 | 0.416 | 0.000 |
| S6 | 0.000 | 0.500 | 0.000 | 0.250 | 0.500 | -1.000 | 1.450 | 0.000 | 0.347 | 0.000 |
| Salambo | 0.483 | 0.450 | 0.152 | 0.198 | 0.300 | -0.416 | 1.550 | 0.235 | 0.302 | 0.200 |
| SalamboS | 0.154 | 0.350 | 0.064 | 0.169 | 0.300 | -0.752 | 1.350 | 0.093 | 0.247 | 0.100 |
| Salanquet | 0.465 | 0.450 | 0.147 | 0.192 | 0.360 | -0.765 | 1.450 | 0.227 | 0.274 | 0.100 |
| SalanquetS | 0.125 | 0.350 | 0.052 | 0.175 | 0.350 | -1.000 | 1.350 | 0.076 | 0.243 | 0.050 |
| Sarramian | 0.301 | 0.750 | 0.102 | 0.303 | 0.348 | -0.133 | 2.000 | 0.154 | 0.481 | 0.850 |
| SErasmo | 0.283 | 0.450 | 0.117 | 0.213 | 0.400 | -0.852 | 1.450 | 0.171 | 0.299 | 0.100 |
| SpinosoPalermo | 0.284 | 0.600 | 0.104 | 0.272 | 0.467 | -0.672 | 1.600 | 0.156 | 0.386 | 0.200 |
| SpinosoSardo | 0.110 | 0.500 | 0.046 | 0.231 | 0.375 | -0.600 | 1.500 | 0.066 | 0.327 | 0.200 |
| SpinosoViolLiguria | 0.162 | 0.450 | 0.067 | 0.219 | 0.425 | -0.926 | 1.450 | 0.098 | 0.305 | 0.100 |
| T31 | 0.000 | 0.450 | 0.000 | 0.225 | 0.450 | -1.000 | 1.350 | 0.000 | 0.312 | 0.050 |
| T32 | 0.000 | 0.450 | 0.000 | 0.225 | 0.450 | -1.000 | 1.300 | 0.000 | 0.312 | 0.050 |
| T33 | 0.000 | 0.450 | 0.000 | 0.225 | 0.450 | -1.000 | 1.450 | 0.000 | 0.312 | 0.050 |
| T34 | 0.000 | 0.500 | 0.000 | 0.250 | 0.500 | -1.000 | 1.500 | 0.000 | 0.347 | 0.050 |
| T35 | 0.000 | 0.450 | 0.000 | 0.225 | 0.450 | -1.000 | 1.450 | 0.000 | 0.312 | 0.000 |
| T36 | 0.000 | 0.550 | 0.000 | 0.275 | 0.550 | -1.000 | 1.550 | 0.000 | 0.381 | 0.000 |
| T37 | 0.000 | 0.550 | 0.000 | 0.275 | 0.550 | -1.000 | 1.550 | 0.000 | 0.381 | 0.000 |
| T38 | 0.000 | 0.550 | 0.000 | 0.275 | 0.550 | -1.000 | 1.550 | 0.000 | 0.381 | 0.050 |
| T39 | 0.000 | 0.550 | 0.000 | 0.275 | 0.550 | -1.000 | 1.550 | 0.000 | 0.381 | 0.100 |
| TeromBA | 0.289 | 0.550 | 0.105 | 0.257 | 0.420 | -0.566 | 1.750 | 0.156 | 0.391 | 0.500 |
| TeromCT | 0.098 | 0.550 | 0.040 | 0.275 | 0.475 | -0.721 | 1.600 | 0.059 | 0.392 | 0.250 |
| TondoPaestum | 0.150 | 0.550 | 0.062 | 0.263 | 0.500 | -0.879 | 1.550 | 0.091 | 0.368 | 0.050 |
| TondoRossoPaestum | 0.444 | 0.600 | 0.137 | 0.277 | 0.500 | -0.707 | 1.600 | 0.210 | 0.391 | 0.050 |
| Velours | 0.424 | 0.600 | 0.137 | 0.291 | 0.530 | -0.767 | 1.600 | 0.210 | 0.406 | 0.100 |
| VerdeCalahorra | 0.336 | 0.650 | 0.115 | 0.315 | 0.275 | 0.218 | 2.100 | 0.173 | 0.519 | 1.150 |
| VerdePeralta | 0.292 | 0.800 | 0.101 | 0.321 | 0.329 | -0.028 | 2.200 | 0.152 | 0.533 | 1.050 |
| VertProvence | 0.478 | 0.700 | 0.148 | 0.329 | 0.610 | -0.805 | 1.750 | 0.229 | 0.469 | 0.200 |
| Vertu | 0.497 | 0.500 | 0.154 | 0.238 | 0.430 | -0.779 | 1.550 | 0.238 | 0.341 | 0.150 |
| VertVaulxVelin | 0.352 | 0.700 | 0.118 | 0.337 | 0.458 | -0.351 | 2.050 | 0.180 | 0.538 | 0.650 |
| VioletCamargue | 0.443 | 0.550 | 0.141 | 0.255 | 0.405 | -0.563 | 1.650 | 0.216 | 0.379 | 0.500 |
| VioletGapeau | 0.465 | 0.550 | 0.149 | 0.263 | 0.490 | -0.841 | 1.550 | 0.228 | 0.369 | 0.000 |
| VioletGapeauI | 0.160 | 0.550 | 0.066 | 0.263 | 0.500 | -0.879 | 1.550 | 0.097 | 0.368 | 0.150 |
| VioletProvence41S | 0.211 | 0.400 | 0.072 | 0.200 | 0.400 | -1.000 | 1.400 | 0.110 | 0.277 | 0.150 |
| VioletProvence45 | 0.280 | 0.600 | 0.116 | 0.288 | 0.550 | -0.889 | 1.600 | 0.169 | 0.403 | 0.150 |
| VioletProvenceF | 0.101 | 0.500 | 0.042 | 0.263 | 0.475 | -0.840 | 1.600 | 0.061 | 0.381 | 0.150 |
| VioletProvenceI | 0.075 | 0.600 | 0.031 | 0.288 | 0.500 | -0.733 | 1.650 | 0.045 | 0.414 | 0.100 |
| ViolettoMaremma | 0.216 | 0.600 | 0.089 | 0.288 | 0.550 | -0.889 | 1.600 | 0.130 | 0.403 | 0.050 |
| ViolettoSicilia10 | 0.107 | 0.650 | 0.044 | 0.306 | 0.525 | -0.692 | 1.650 | 0.065 | 0.431 | 0.150 |
| ViolettoSicilia13 | 0.062 | 0.500 | 0.026 | 0.238 | 0.450 | -0.867 | 1.500 | 0.037 | 0.333 | 0.050 |
| ViolettoSicilia14 | 0.152 | 0.500 | 0.063 | 0.244 | 0.475 | -0.933 | 1.500 | 0.092 | 0.340 | 0.050 |
| ViolettoSicilia3 | 0.234 | 0.550 | 0.097 | 0.269 | 0.525 | -0.939 | 1.550 | 0.142 | 0.375 | 0.050 |
| ViolettoSicilia4 | 0.116 | 0.450 | 0.048 | 0.225 | 0.450 | -1.000 | 1.450 | 0.070 | 0.312 | 0.100 |
| ViolettoSicilia61 | 0.110 | 0.450 | 0.046 | 0.219 | 0.425 | -0.926 | 1.450 | 0.066 | 0.305 | 0.000 |
| ViolettoSicilia64 | 0.084 | 0.550 | 0.035 | 0.269 | 0.525 | -0.939 | 1.550 | 0.051 | 0.375 | 0.050 |
| ViolettoSicilia98 | 0.164 | 0.600 | 0.068 | 0.288 | 0.500 | -0.722 | 1.600 | 0.099 | 0.403 | 0.200 |
| ViolettoSiciliab3 | 0.083 | 0.600 | 0.034 | 0.281 | 0.525 | -0.833 | 1.600 | 0.050 | 0.396 | 0.050 |
| ViolettoSiciliai2 | 0.103 | 0.550 | 0.043 | 0.275 | 0.550 | -1.000 | 1.550 | 0.062 | 0.381 | 0.000 |
| ViolettoSiciliai3 | 0.419 | 0.550 | 0.133 | 0.277 | 0.490 | -0.775 | 1.650 | 0.205 | 0.400 | 0.050 |
| ViolettoToscana | 0.156 | 0.550 | 0.065 | 0.263 | 0.500 | -0.879 | 1.500 | 0.094 | 0.368 | 0.100 |
| ViolProvence41I | 0.116 | 0.600 | 0.048 | 0.288 | 0.500 | -0.722 | 1.600 | 0.070 | 0.403 | 0.050 |
| ViolProvence45I | 0.453 | 0.700 | 0.141 | 0.314 | 0.530 | -0.608 | 1.750 | 0.218 | 0.454 | 0.150 |
| ViolProvence73 | 0.384 | 0.750 | 0.151 | 0.340 | 0.490 | -0.384 | 1.900 | 0.221 | 0.506 | 0.300 |
| ViolProvenceV | 0.087 | 0.500 | 0.036 | 0.244 | 0.475 | -0.933 | 1.500 | 0.053 | 0.340 | 0.050 |
| ViolProvenceVM | 0.121 | 0.600 | 0.050 | 0.288 | 0.550 | -0.889 | 1.600 | 0.073 | 0.403 | 0.050 |
| ViolProvenceVP | 0.103 | 0.650 | 0.043 | 0.319 | 0.575 | -0.795 | 1.650 | 0.062 | 0.444 | 0.150 |
| ViolProvenceVPG | 0.124 | 0.650 | 0.051 | 0.313 | 0.600 | -0.897 | 1.610 | 0.075 | 0.000 | 0.400 |
| ViolProvenceVR | 0.188 | 0.650 | 0.078 | 0.313 | 0.600 | -0.897 | 1.610 | 0.114 | 0.000 | 0.350 |
| Mean | 0.20 | 0.54 | 0.07 | 0.257 | 0.448 | -0.737 | 1.58 | 0.11 | 0.36 | 0.19 |
