# Supplementary material for: Genetic diversity and accession structure in European Cynara cardunculus collections
Source: PLoS One. 2017 Jun 1;12(6):e0178770. doi: 10.1371/journal.pone.0178770 (PMC5453587; doi:10.1371/journal.pone.0178770)
Supplement: S7 Table — Number of alleles (Na), Number of effective alleles (Ne), Shannon's Information Index (I), expected heterozygosity (He), Unbiased Expected Heterozygosity (UHe), and amplicons in the case of dominant markers. (DOCX) [file pone.0178770.s009.docx]

S7 Table. Average value per accession for each markers used to genotype *Cynara cardunculus* individuals. Number of alleles (Na), Number of effective alleles (Ne), Shannon's Information Index (I), expected heterozygosity (He), Unbiased Expected Heterozygosity (UHe), and amplicons in the case of dominant markers.

| **Marker** | **Na** | **Ne** | **I** | **He** | **UHe** | **Amplicons** |
| --- | --- | --- | --- | --- | --- | --- |
| EaccMcta | 0.839 | 1.039 | 0.034 | 0.023 | 0.027 | 32 |
| EacgMctt | 0.728 | 1.065 | 0.058 | 0.038 | 0.042 | 44 |
| EagcMctt | 0.775 | 1.035 | 0.029 | 0.020 | 0.023 | 35 |
| MacPca | 0.666 | 1.152 | 0.133 | 0.089 | 0.107 | 192 |
| MacPcg | 0.610 | 1.141 | 0.125 | 0.083 | 0.100 | 154 |
| MgcPca | 0.610 | 1.132 | 0.119 | 0.079 | 0.094 | 167 |
| MgcPcg | 0.591 | 1.126 | 0.115 | 0.076 | 0.091 | 127 |
| 810 | 0.514 | 1.128 | 0.118 | 0.078 | 0.095 | 10 |
| 818 | 0.583 | 1.011 | 0.008 | 0.006 | 0.006 | 14 |
| 827 | 0.476 | 1.013 | 0.011 | 0.007 | 0.009 | 14 |
| 834 | 0.403 | 1.113 | 0.099 | 0.067 | 0.085 | 6 |
| 840 | 0.675 | 1.010 | 0.009 | 0.006 | 0.007 | 13 |
| 841 | 0.490 | 1.120 | 0.110 | 0.073 | 0.088 | 47 |
| 855 | 0.458 | 1.016 | 0.014 | 0.010 | 0.011 | 10 |
| 857 | 0.472 | 1.111 | 0.107 | 0.069 | 0.085 | 71 |
| 857c | 0.521 | 1.028 | 0.024 | 0.016 | 0.018 | 14 |
| 857g | 0.710 | 1.025 | 0.022 | 0.015 | 0.016 | 10 |
| 872 | 0.422 | 1.005 | 0.004 | 0.003 | 0.003 | 14 |
| CsCiCaCa05 | 2.000 | 1.557 | 0.497 | 0.319 | 0.357 |  |
| CDAT-01 | 2.071 | 1.844 | 0.609 | 0.407 | 0.453 |  |
| CLIB-02I | 1.571 | 1.390 | 0.323 | 0.220 | 0.244 |  |
| CLIB-02II | 2.071 | 1.680 | 0.547 | 0.349 | 0.387 |  |
| CLIB-12 | 1.571 | 1.161 | 0.201 | 0.114 | 0.127 |  |
| CMAFLP-01 | 1.857 | 1.509 | 0.434 | 0.281 | 0.328 |  |
| CMAFLP-04 | 1.286 | 1.163 | 0.301 | 0.185 | 0.223 |  |
| CMAFLP-05 | 1.000 | 1.000 | 0.000 | 0.000 | 0.000 |  |
| CMAFLP-18 | 3.000 | 2.170 | 0.876 | 0.517 | 0.583 |  |
| CMAL06 | 2.786 | 2.103 | 0.772 | 0.445 | 0.500 |  |
| CMAL-108 | 1.429 | 1.236 | 0.209 | 0.134 | 0.149 |  |
| CMAL11 | 1.857 | 1.560 | 0.471 | 0.318 | 0.357 |  |
| CMAL117 | 2.071 | 1.468 | 0.458 | 0.278 | 0.312 |  |
| CMAL21 | 3.286 | 2.550 | 0.934 | 0.513 | 0.574 |  |
| CMAL24 | 1.286 | 1.210 | 0.171 | 0.117 | 0.129 |  |
| CMAL-25 | 2.000 | 1.968 | 0.683 | 0.490 | 0.548 |  |
| CsPal02 | 2.143 | 1.809 | 0.596 | 0.379 | 0.425 |  |
| CsPal03 | 1.786 | 1.514 | 0.415 | 0.270 | 0.303 |  |
| CsEST03 | 2.571 | 1.891 | 0.658 | 0.380 | 0.425 |  |
| FA2-GAT | 1.714 | 1.547 | 0.421 | 0.292 | 0.329 |  |
